# Altered Rich-Club and Frequency-Dependent Subnetwork Organization in Mild Traumatic Brain Injury: A MEG Resting-State Study

**DOI:** 10.3389/fnhum.2017.00416

**Published:** 2017-08-30

**Authors:** Marios Antonakakis, Stavros I. Dimitriadis, Michalis Zervakis, Andrew C. Papanicolaou, George Zouridakis

**Affiliations:** ^1^Institute of Biomagnetism and Biosignal Analysis, Westfalian Wilhelms-University Muenster Muenster, Germany; ^2^Digital Image and Signal Processing Laboratory, School of Electronic and Computer Engineering, Technical University of Crete Chania, Greece; ^3^Institute of Psychological Medicine and Clinical Neurosciences, Cardiff University School of Medicine Cardiff, United Kingdom; ^4^Cardiff University Brain Research Imaging Center (CUBRIC), School of Psychology, Cardiff University Cardiff, United Kingdom; ^5^Neuroinformatics Group, Cardiff University Brain Research Imaging Center (CUBRIC), School of Psychology, Cardiff University Cardiff, United Kingdom; ^6^School of Psychology, Cardiff University Cardiff, United Kingdom; ^7^Departments of Pediatrics, and Anatomy and Neurobiology, Neuroscience Institute, University of Tennessee Health Science Center, Le Bonheur Children's Hospital Memphis, TN, United States; ^8^Biomedical Imaging Lab, Departments of Engineering Technology, Computer Science, Biomedical Engineering, and Electrical and Computer Engineering, University of Houston Houston, TX, United States

**Keywords:** magnetoencephalography (MEG), mild traumatic brain injury, network resilience, cross-frequency coupling, intrinsic networks

## Abstract

Functional brain connectivity networks exhibit “small-world” characteristics and some of these networks follow a “rich-club” organization, whereby a few nodes of high connectivity (hubs) tend to connect more densely among themselves than to nodes of lower connectivity. The Current study followed an “attack strategy” to compare the rich-club and small-world network organization models using Magnetoencephalographic (MEG) recordings from mild traumatic brain injury (mTBI) patients and neurologically healthy controls to identify the topology that describes the underlying intrinsic brain network organization. We hypothesized that the reduction in global efficiency caused by an attack targeting a model's hubs would reveal the “true” underlying topological organization. Connectivity networks were estimated using mutual information as the basis for cross-frequency coupling. Our results revealed a prominent rich-club network organization for both groups. In particular, mTBI patients demonstrated hyper-synchronization among rich-club hubs compared to controls in the δ band and the δ-γ_1_, θ-γ_1_, and β-γ_2_ frequency pairs. Moreover, rich-club hubs in mTBI patients were overrepresented in right frontal brain areas, from θ to γ_1_ frequencies, and underrepresented in left occipital regions in the δ-β, δ-γ_1_, θ-β, and β-γ_2_ frequency pairs. These findings indicate that the rich-club organization of resting-state MEG, considering its role in information integration and its vulnerability to various disorders like mTBI, may have a significant predictive value in the development of reliable biomarkers to help the validation of the recovery from mTBI. Furthermore, the proposed approach might be used as a validation tool to assess patient recovery.

## Introduction

Mild traumatic brain injury (mTBI) is a significant cause of brain insult (Len and Neary, 2011; Huang et al., 2014) representing close to 90% of all brain injuries (Len and Neary, 2011). Approximately 5–20% of the irremediable patients (Bharath et al., 2015) still suffer from post-concussion symptoms several months after the initial injury (Huang et al., 2014). These symptoms are often characterized by physical, emotional, cognitive, and sleep disturbances and may take many months to return to the baseline (Huang et al., 2014). Several neuropsychological studies have reported reduced cognitive efficiency, especially in tests measuring executive function, processing speed, attention, connectivity, and memory in mTBI patients with persistent symptoms (Huang et al., 2014; Pang et al., 2016). Management of mTBI is crucial due to its deleterious effects on certain brain functions, including attention (De Monte et al., 2006), working memory (Vanderploeg et al., 2005), and verbal learning (De Monte et al., 2006).

The human brain can be viewed as a neurophysiological network of brain areas that are structurally and functionally interconnected. These distinct networks are temporally and spatially distributed and exist in a range of spatiotemporal scales. Spatially, they extend from microscopic networks of single neurons and local synaptic interactions to large-scale brain networks interconnected via long white-matter tracts (Eierud et al., 2014). The time domain scales vary from milliseconds to seconds (Dimitriadis et al., 2013a, 2015a; Hansen et al., 2015; Betzel et al., 2016). Considering that interactions among large-scale networks are significant for high-level cognitive functions, most recent functional connectivity (FC) studies of mTBI focus on large-scale intrinsic connectivity networks (ICNs) aiming at identifying the changes they undergo as a result of injury. FC is reflected in the neurophysiological activity of neural populations that mediate cortical communication and information integration (Wang, 2010). Clinically, FC has been shown useful in the study of several neurological and neuropsychiatric disorders and their symptoms (Tewarie et al., 2013, for a review see Eierud et al., 2014).

During the past several years, numerous studies, including ours, have attempted to develop reliable biomarkers of mTBI based on resting state MEG. While other imaging modalities measure brain activation indirectly, such as functional Magnetic Resonance Imaging (fMRI) which relies on hemodynamic events, MEG measures neuronal activity directly. MEG possesses most of the highly desired neuroimaging properties, including high sensitivity and efficient handling of environmental noise, and combines very good spatial details with excellent temporal resolution (Dimitriadis et al., 2013b; Antonakakis et al., 2015, 2016). Numerous analysis techniques applied to EEG and MEG recordings have clearly demonstrated altered functional connectivity in TBI that is closely correlated with disease severity (Castellanos et al., 2010). Zouridakis et al. (2012) using Granger causality showed that mTBI patients exhibited a sparsely distributed network of long-range connections compared to controls during the first few weeks after mTBI. Tarapore et al. (2013) found that resting state MEG could detect abnormal connectivity in TBI, while Da Costa et al. (2015) suggested that MEG could detect subtle neural changes associated with cognitive dysfunction in mTBI. Huang et al. (2014) uncovered anatomical and functional correlations between abnormal slow waves and mild axonal injury. Dunkley et al. (2015) found increased connectivity in mTBI that was limited to slow wave amplitude coupling. Dimitriadis et al. (2015b) used phase-locking value estimates to quantify intra-frequency couplings and showed that this pattern was mostly seen in the delta band, whereas Antonakakis et al. (2015, 2016), in a follow-up analysis based on inter-frequency couplings, showed that controls formed a dense network of stronger local and global connections in agreement with other studies (Rapp et al., 2015).

Among the various procedures to estimate FC networks, also known as FC graphs (FCG), intra-frequency measures, such as mutual information (MI) (Bullmore and Bassett, 2011; Tsiaras et al., 2011), and inter-frequency measures, such as cross-frequency coupling (CFC) (Antonakakis et al., 2015, 2016; Dimitriadis et al., 2015c; Florin and Baillet, 2015), are of special interest in the topological analysis of FCGs.

Several brain connectivity studies using fMRI and electro- (EEG) and magneto- (MEG) encephalography have suggested that FCGs exhibit properties of a “small world” (SW) network organization (or simple organization) (Micheloyannis et al., 2006; Palva and Palva, 2011; Dimitriadis et al., 2015a; Vértes and Bullmore, 2015). SW is a special type of mathematical graph in which the majority of the nodes are not direct neighbors; yet, most nodes can be reached from any other node within a small number of steps. Thus, SW networks are simultaneously highly clustered and highly efficient. In particular, when considered as nodes that are connected to each other, SW networks are likely to have many first degree neighbors in common, and the average path length between a pair of nodes is short (Vértes and Bullmore, 2015).

An additional property often seen in SW organization is the formation of certain nodes (termed “hubs”) that are more densely connected to each other than the rest of the nodes (Van den Heuvel and Sporns, 2011). Hubs with the highest interconnectivity values follow what is known as “rich club” (RC) organization and appear to be the most relevant nodes in a network in terms of global information processing (Van den Heuvel and Sporns, 2011; Van den Heuvel et al., 2013; Schroeter et al., 2015; Vértes and Bullmore, 2015). Thus, the RC organization can be seen as a variation of the SW network organization, with different topological properties (Mišić et al., 2014) featuring disproportionately dense interconnections (Van den Heuvel and Sporns, 2011; Bullmore and Sporns, 2012; Mišić et al., 2014). These hubs support more traffic than the ordinary SW nodes (Mišić et al., 2014), and effectively define the top-level structure of a network, its hierarchical ordering, and node specialization (Van den Heuvel and Sporns, 2011).

Typically, the largest amount of information flow between pairs of nodes in human brain networks passes through RC and SW hubs (Palva and Palva, 2011; Van den Heuvel and Sporns, 2011; Vértes and Bullmore, 2015). As a consequence, the SW organization has been studied in several brain disorders, including schizophrenia (Micheloyannis et al., 2006), Alzheimer's disease (Stam et al., 2007), autism (Rubinov and Sporns, 2010; Tsiaras et al., 2011), and epilepsy (Bharath et al., 2016). The RC networks have been explored in brain ischemia (Van den Heuvel et al., 2013; Watanabe and Rees, 2015; Crossley et al., 2016), in healthy subjects using diffusion tensor imaging MRI (Van den Heuvel and Sporns, 2011) and recently in studies of brain activation (Van den Heuvel and Sporns, 2011; Bullmore and Sporns, 2012; Mišić et al., 2014; Antonakakis et al., 2015) whereby RC hubs were shown to play a pivotal role in information integration.

Given our previous use of connectivity analysis to study mTBI using Granger causality (Zouridakis et al., 2012), phase synchronization (Dimitriadis et al., 2015b), and cross-frequency coupling (Antonakakis et al., 2015, 2016) of spontaneous MEG, as well as brain activation patterns of both EEG and MEG at the sensor (Li et al., 2015) and source (Zouridakis et al., 2016; Li et al., 2017) levels, an obvious question is whether the possible presence of an RC organization could provide some complementary features to the SW organization that is typically seen in mTBI FCGs. Thus, in the present study, we hypothesize that exploring brain connectivity network models derived from spontaneous MEG activity using estimators for both intra and cross-frequency couplings (Buzsáki and Watson, 2012) would help identify meaningful network topological features in compromised mTBI brain networks that could be used as guideline biomarkers for validating the recovery from mTBI (Bharath et al., 2015). For a better understanding of network topologies linked to mTBI, we followed an attack strategy to reveal the key network model, either small-world or rich-club, that best describes mTBI functional brain networks. Our network analysis was based on intra- inter-frequency, and integrated functional brain networks to cover the different aspects of the multiplexity of brain rhythms via brain connectivity.

The SW and RC organizations, however, are not mutually exclusive, considering that an RC network may also present SW characteristics in a subnetwork (Bullmore and Sporns, 2012). In particular, subareas of an RC network can simultaneously be part of a SW network featuring SW behavior. Thus, to identify the network organization that explains best the network topology of intra-frequency and inter-frequency FCGs, for both normal controls and mTBI patients, we implemented an “attack strategy” (Van den Heuvel and Sporns, 2011; Antonakakis et al., 2015) on SW and RC nodes to reveal their relative importance in information transfer and neural communication within the entire brain network. The present study is an extension of our recent short report (Antonakakis et al., 2015) which compared the RC and SW organizations only in the δ-β cross-frequency pair and found that resting state MEG FCGs followed a rich-club organization.

## Materials and methods

### Participants and recording procedure

Thirty right-handed individuals (29.33 ± 9.2 years of age) with mTBI (Levin, 2009; Dimitriadis et al., 2015b) and fifty age- and gender-matched neurologically intact controls (29.25 ± 9.1 years of age) were analyzed. The appropriate review boards at participating institutions approved all procedures and all subjects provided informed consent and they had agreed verbally and writtenly. This work was approved by the Institutional Review Boards (IRBs) and the Human Research Protections Official's (HRPO) Review of Research and Protocols for the Department of Defense. All procedures were compliant with the Health Insurance Portability and Accountability Act (HIPAA). A subject was identified as mTBI based on clinical evaluation and head injury occurring within 24 h. Furthermore, exclusion criteria included existence of previous disease, high blood alcohol level, and score on the Abbreviated Injury Scale and other parameters that are described in Section 8 of the Supplementary Material.

Approximately 3–5 minutes of eyes-closed, resting-state MEG activity was recorded from each subject, using a 248-channel Magnes WH3600 system (4D Neuroimaging Inc., San Diego, CA). Data were collected at a sampling rate of 1,017.25 Hz. Axial gradiometer recordings were transformed to planar gradiometer field approximations using the “sincos” method of Fieldtrip (Oostenveld et al., 2011). The raw MEG data were preprocessed by means of ICA (see Section 1 of Supplementary Material).

## Elements of graph theory

### Types of functional connectivity graphs—FCG

To investigate different types of networks, artifact-free multidimensional arrays of time series X were filtered in several frequency bands (f), namely δ (0.5–4 Hz), θ (4–8 Hz), α (8–15 Hz), β (15–30 Hz), γ_1_(30–45 Hz), and γ_2_(45–80 Hz), creating a single multidimensional array *X*_*f*_ for each subject.

We also explored intra-frequency connectivity graphs (undirected IFCGs), cross-frequency connectivity graphs (directed CFCGs), and a combination of both IFCGs and CFCGs, which we called intra-cross-frequency connectivity graphs (ICFCG). The IFCG were constructed using Mutual Information (MI), a nonlinear metric that can reveal synchronization between time series from different sensors in a particular frequency band. Therefore, MI uncovers the interdependence among the MEG sensors and simultaneously expresses the intra-frequency content between two time series within a brain rhythm. In addition, we explored CFCGs using cross-frequency interactions and phase-to-amplitude couplings (PACs), whereby the phase of a low-frequency rhythm could modulate the amplitude of a higher-frequency oscillation (Antonakakis et al., 2016). Cross-frequency coupling (CFC) is thought to represent a basic mechanism of functional integration of neural networks across distant brain regions (i.e., the inter-frequency content of brain rhythms). Moreover, the ICFCG, the new type of FCG, was designed to quantify the maximum interaction between the two types of FCG and was constructed by the most dominant connections (either IFCG or CFCG) among the frequency pairs and frequency bands for each pair of MEG sensors.

### Intra-frequency connectivity graphs—IFCG

IFCG were constructed based on mutual Information (MI), which measures the interdependence of two time series *X*_*f,i*_ and *X*_*f,j*_, with *i,j* = 1 …248, that are part of the multidimensional array *X*_f_. MI stems from information theory and offers several advantages compared to other measures, such as sensitivity to any type of dependence between the time series, including nonlinear relations and generalized synchronization, robustness to outliers, and measurement in information bits. The mathematical definition of MI between two artifact-free sensor datasets *X*_f,i_ and *X*_f,j_, filtered in the specific frequency bands *f*_i_ and *f*_j_, is given by

(1) $$IFCG_{f}\left(i,j\right)=I\left(X_{f,i};X_{f,j}\right)=\sum_{y\in Y}\sum_{z\in Z}p\left(z,y\right)\log\left(\frac{p\left(z,y\right)}{p_{z}\left(z\right)p_{y}\left(y\right)}\right)$$

where *Z* = *X*_*f,i*_, *Y* = *X*_*f,j*_, *p*(z,y) is the joint probability distribution function of *Z* and *Y*, respectively, and *p*_*z*_(*z*) = ∑_*y*∈*Y*_ *p*(*z*, *y*) and [*p*_*y*_(*y*) = ∑_*z*∈*Z*_ *p*(*z*, *y*)] are the marginal probability distribution functions of *Z* and *Y*, respectively (Tsiaras et al., 2011; Antonakakis et al., 2015, 2016).

### Cross-frequency connectivity graph—CFCG

In the case of CFCG with f_c_ = (δ, θ), …, (γ_1_, γ_2_), we explored cross-frequency interactions using PAC, whereby the phase of a low-frequency (f_l_) rhythm modulated the amplitude of a higher-frequency (f_h_) oscillation (Tsiaras et al., 2011; Antonakakis et al., 2015, 2016). Furthermore, for each pair of time series stremming from sensors {i,j}, we estimated intra- and inter-frequency coupling using MI. Based on surrogate data analysis and on false discovery rate (FDR) correction to account for multiple comparisons (Benjamini and Hochberg, 1995), we assigned one dominant type of interaction for each pair of sensors (Dimitriadis et al., 2016). The mathematical aspects of PAC estimation, the surrogate data analysis that we followed for estimating intra- and inter-frequency couplings, and the estimation of the dominant type of coupling are described in Section 2 of Supplementary Material.

### Intra-cross-frequency connectivity graphs—ICFCG

Beyond the above FCGs, an additional FCG type was estimated to quantify combined intra- and inter-frequency couplings. The ICFCGs were estimated by combining all frequency pairs (15 frequency pairs) and frequencies (6 frequencies) for each pair of sensors. The mathematical definition of this type of FCG is given by the following equation,

(2) $$\begin{array}{l} ICFCG\left(i,j\right)=\underset{\begin{array}{ccc} fc & = & \left(\delta ,\theta \right),\ldots ,\left(\gamma_{1},\gamma_{2}\right)\\ f & = & \left(\delta ,\ldots ,\gamma_{1}\right) \end{array}}{\max}\;\left\{IFCG_{f}\left(i,j\right),CFCG_{fc}\left(i,j\right)\right\}\forall \\ \;i,j=1,\ldots ,248 \end{array}$$

The detailed description of how ICFCGs were defined is given in Section 3 of Supplementary Material. Briefly, we employed surrogate data analysis to identify significant intra- and cross-frequency interactions that were estimated for all predefined frequency bands and frequency pairs, within and between all 248 MEG sensors.

### Topological filtering of FCG

After applying surrogate analysis to IFCGs, CFCGs, and ICFCGs to extract the significant connections, we employed a data-driven topological filtering approach to uncover the connections that optimized the global information flow constrained to the cost of the selected connections (see Section 4 of Supplementary Material).

### Classification of functional connectivity patterns

To evaluate whether the ICFCG defined a characteristic graph for each of the two groups, we examined the prediction power of these graph structures and developed a classification scheme similar to our previous studies (Antonakakis et al., 2015, 2016; Dimitriadis et al., 2015b). Specifically, classification of ICFCGs from individual subjects started by performing tensor space analysis (TSA)^1^ (Dimitriadis et al., 2015b), which was followed by comparison with FCGs of known labels. We adopted three different classification schemes, namely TSA with k nearest neighbor (kNN) classification (TSA+kNN), TSA with ensemble classification (TSA+ENS), and TSA with extreme learning machine (ELM) classification (TSA+ELM). In particular, the number of neighbors, k, was selected based on the best accuracy obtained by iterating the TSA+kNN classification scheme with k varying from 5 to 20. To compare the performance of the ICFCGs, we also created a multilayer graph that included IFCGs and CFCGs and evaluated the same classification schemes. The description of these schemes and their performance evaluation is given in Section 5 of the Supplementary Material. Finally, the edges of each FCG were filtered out to reduce the total number of connection. The approach is described in Section 4 of Supplementary Material.

### Validating brain models via network attacks

#### Small world and rich club organizations

We estimated SW network organization based on weighed versions of global efficiency (GE) and local efficiency (LE) (Latora and Marchiori, 2001) for each type of FCG, directed or undirected (IFCG, CFCG, and ICFCG). In addition to SW, the RC organization was computed based on the distribution of the node degree and the weights of every type of FCG (Antonakakis et al., 2015) (see Section 6 of Supplementary Material).

#### Network attacks

The attack strategy focused on SW or RC nodes to reveal their importance in information transfer in the whole network. RC subnetworks as part of an overall network have a strong positive impact on the GE of the whole structure (Van den Heuvel and Sporns, 2011). Thus, the role of a node, or a set of nodes, in terms of network GE could be evaluated by examining the “damage” inflicted on that node by an attack, simulated as a decrease in the weights of its connections (Van den Heuvel and Sporns, 2011; Antonakakis et al., 2015). In particular, two forms of attack were distinguished: “targeted attack” and “random attack” to hub connections.

In the targeted attack, a set of connections (either 50 or 100%) within interconnected RC (target RC or TRC) or SW (target SW or TSW) nodes were randomly selected and attacked at two levels of severity, inflicting 50 or 100% damage, respectively, to the weights of all connections. In the random attack, we restricted the damage to the subset of the connections between hub RC (hubs, random RC or HRRC) or hub SW (hubs, random SW or HRSW) nodes and the rest of the network. Similar to the target attack, damage was inflicted by reducing the weights of the connections of the selected random set (50 and 100%) by 50 or 100%. For each attack strategy, we randomly selected 50 and 100% of each type of connection and then their weights were damaged by 50 and 100%. Each condition (2 levels of randomly selected subsets of connections × 2 levels of damage of weight connections = 4) was repeated 1,000 times for the RC and SW models. Then, the two topologies were compared assuming that the smallest reduction in global %GE was the best fit for the “real” underlying brain network. In particular, we estimated the effect of a reduction in %GE of the network following a targeted attack on SW or RC connections against the effect of a random attack to hub connections. To reduce the bias due to the different number of nodes in the two architectures, we selected a subset of RC nodes equal to the number of SW nodes for all four possible attack cases. Then, to build meaningful statistics, we considered 100 different subsets of RC nodes. Finally, we averaged the %GE across all subsets of RC nodes from the 1,000 iterations and across the 100 distinct subsets and tested for statistical differences using the statistical analysis described below that was also used in our published studies (Dimitriadis et al., 2013b, 2015c; Antonakakis et al., 2015, 2016).

#### Exploration of difference through statistical analysis

**Figure 1:**
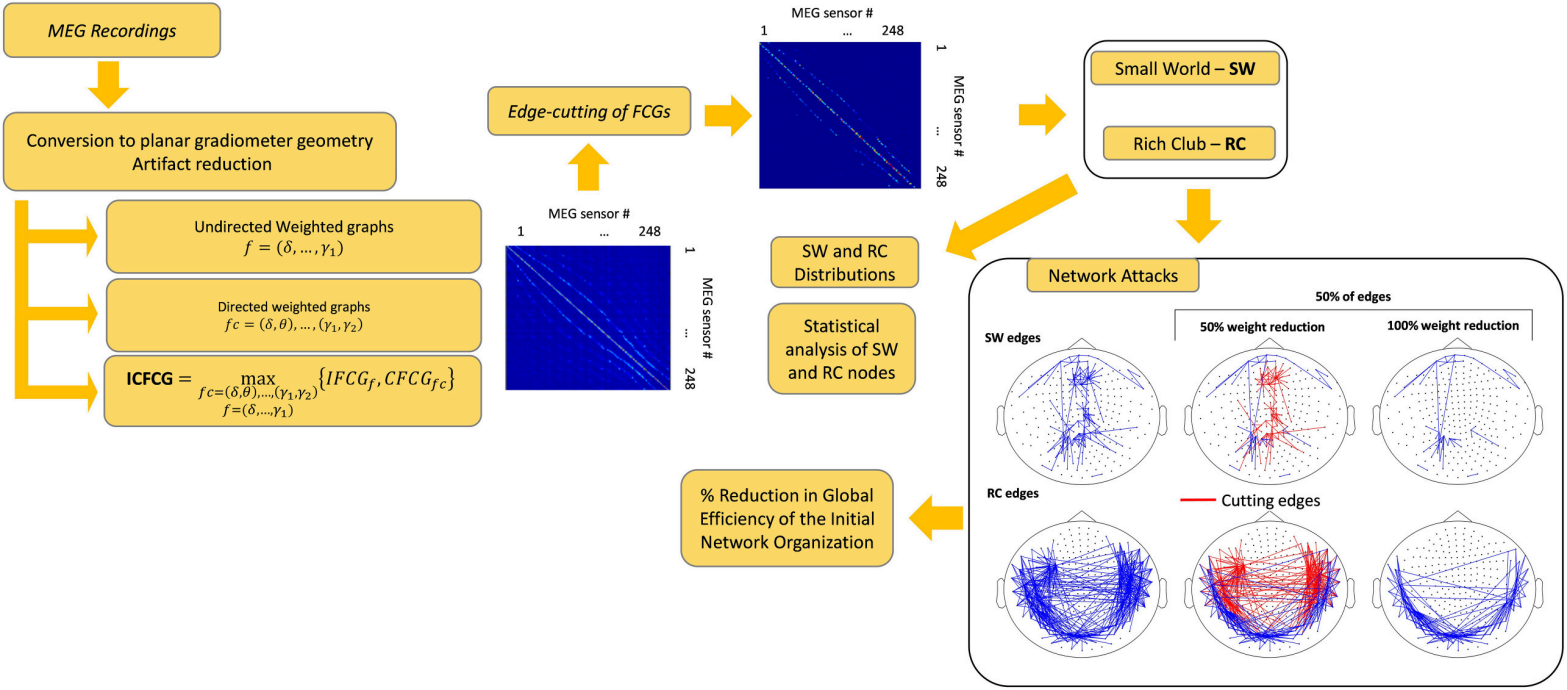
A brief outline of the proposed analysis procedure.

Statistical analysis was performed in all comparisons between the two network organizations and between the two groups. The statistical methods used included testing for normality as well as parametric and non-parametric pair-wise tests (Antonakakis et al., 2016). The threshold for significance of the *p*-value was set to 95%. After FDR adjustment (Benjamini and Hochberg, 1995) the new *p*' values where given by *p*' = *p*/number of cases, where cases was either the number of frequencies for the IFCG or the number of frequency pairs for the IFCG. Figure 1 summarizes the three main steps of the proposed procedure necessary to obtain the FCGs and their topological parameters.

#### Group differences in hub distribution in RC and SW brain network models

We explored group differences in terms of probability distribution of RC or SW hubs in different brain areas and all intra- and cross-frequency cases using the Wilcoxon rank-sum test of discrete probability (DP) to compare the two groups (*p* < 0.01, Bonferroni corrected *p*' = *p*/10, where 10 is the number of brain areas).

#### Group spatial distribution for network organization

In an attempt to represent the spatial distribution of RC and SW hubs over each group consistently, we integrated their representation over different brain areas (frontal, central, temporal, parietal, and occipital) in both hemispheres. In particular, we measured the discrete probability (DP) separately for RC and SW hubs across brain regions and independently for each subject, as the ratio of the number of SW or RC nodes in a specific brain area to the total number of SW or RC nodes detected for that subject. The value of DP ranged from 0 to 1 and the summation of all sub-probabilities for each subject was one. RC or SW hubs were kept as 1s in a 1D vector Hub {1, 248}, where the 248 positions were equal to the number of MEG sensors,

(3) $$DP_{for\;each\;subject}=\frac{\sum_{k\;=1}^{sensors^{lobe}}SW\;or\;RC\;Hub}{\sum_{k\;=1}^{sensors}SW\;or\;RC\;Hub}.$$

#### Estimation of the level of synchronization within the RC subnetwork

The level of synchronization within an RC subnetwork was estimated using the strength ratio (SR), which was defined as the ratio of the strength of the within-interconnected RC nodes to the strength of the sub-network composed of links between RC nodes and the rest of the network. Mathematically SR was defined as follows,

(4) $$SR=\frac{\sum_{k\;=1}^{sensors^{Hubs}}\sum_{k\;=1}^{sensors^{Hubs}}SW\;or\;RC\;Hubs}{\sum_{k\;=1}^{sensors}\sum_{k\;=1}^{sensors}SW\;or\;RC\;Hubs}.$$

## Results

### Classification performance of the ICFCG

Table 1 summarizes the classification performance of ICFCGs in terms of accuracy, sensitivity, and specificity. The control subjects were assigned positive labels and the mTBI patients negative ones. The TSA+kNN combination showed classification accuracy >90%, while the TSA+ENS and TSA+ELM combinations showed similar but somewhat lower performances. In particular, the highest sensitivity (~95%), specificity (~85%), and overall accuracy (~92%) were obtained with the kNN algorithm, whereas the other classification schemes showed ~89% sensitivity and ~81% specificity, respectively. For the kNN results, the best number of neighbors was *k* = 5, based on the mean accuracy obtained for *k* = 5–20. For comparison purposes, the alternative classification scheme using the multi-layer graph showed lower classification accuracy (89.5%), as shown in Table S1.

**Table 1:** Summary of ICFCGs classification performance based on 10-fold cross-validation of TSA for feature selection and kNN, ENS, ELM for classification.

**Classification scheme**	**Accuracy (%)**	**Sensitivity (%)**	**Specificity (%)**
TSA+kNN	91.5 ± 2.2	95.4 ± 3	85 ± 2.4
TSA+ENS	84.5 ± 3.64	89.64 ± 3.58	82.95 ± 4.76
TSA+ELM	83.69 ± 3.84	88.45 ± 4.58	80.99 ± 4.66

### Group spatial distribution for network organization

**Figure 2:**
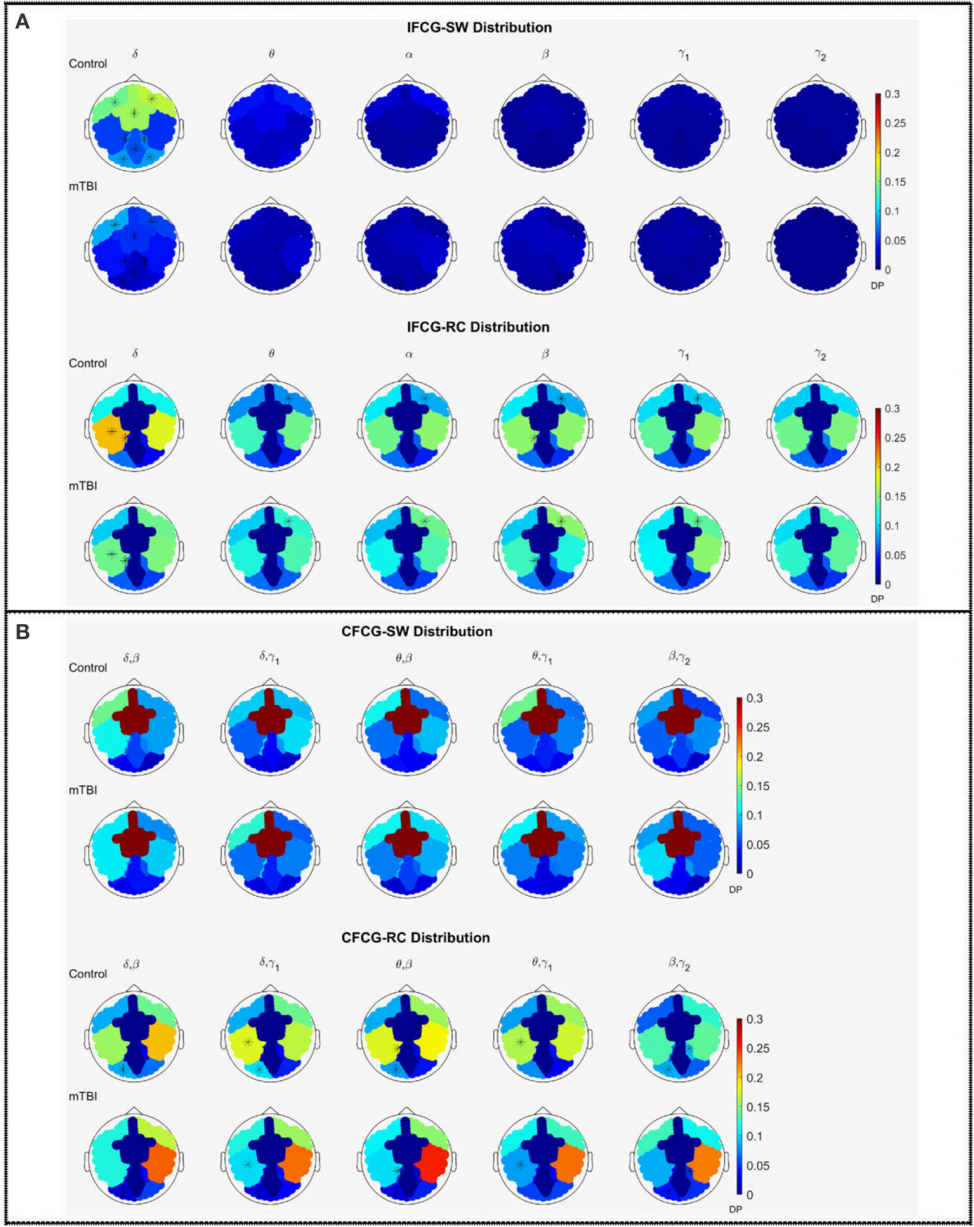
Average distribution of SW and RC nodes on the head surface for control and mTBI subjects showing **(A)** intra-frequency (MI-IFCG) and **(B)** inter-frequency (CFC-CFCG) connections for each FCG type. In **(B)** only the top five cross-frequency pairs with the highest classification accuracy are shown. The colorbar is common type of FCG and network organization represents discrete probability (DP).

The average discrete probability (DP) distributions obtained are depicted in Figure 2 for both FCG types (IFCG or CFCG), groups (Control or mTBI), and network organization (SW or RC). Statistical analysis was performed for each network organization and type of FCG to reveal differences between the two groups.

In the IFCG-SW distribution of controls (Figure 2A, upper part), most of the SW nodes were located in frontal and central areas for all frequency bands and in right temporal region for the γ_1_ and γ_2_ bands. Apart from a higher probability seen in the δ band, the rest of the bands, θ to γ_2_, showed low probabilities, indicating that the SW organization involved a small number of nodes. In the mTBI group, the IFCG-SW distribution included the left frontal, right temporal, and central brain regions which showed the highest probabilities in all bands, from α to γ_1_(Figure 2A, upper part). Significant differences between the two groups based on the SW topology were observed in most of areas expect temporal areas in δ frequency band (Figure 2A, upper part). The IFCG-RC topology showed hubs mostly in temporal areas bilaterally for all frequency bands (Figure 2A, lower part) in both groups. The IFCG-RC distribution showed significant differences in the left temporal, left parietal, and right frontal regions in the δ band and the θ to γ_1_ frequencies, respectively (Figure 2A, lower part).

On the other hand, the CFCG-SW organization revealed a tendency for cross-frequency differences (Figure 2B, upper part) with higher probabilities over central areas in all frequency pairs but without any statistically significant differences between the two groups. When comparing the CFCG-SW organization (Figure 2B, upper part) with its intra-frequency counterpart (Figure 2A, upper part), the observed tendency seemed to be due to the obvious differences in central areas among different bands observed in the IFCG-SW distributions for both groups (Figure 2A, upper part).

Finally, we compared RC distribution within and between frequency bands. The CFCG-RC distribution mostly followed the distribution structure of the IFCG-RC with the highest values mainly in the temporal areas. Finally, the statistically significant group-differences based on CFCG-RC were mostly located in left occipital regions for all frequency pairs except for the θ-γ_1_ frequency couple; which was localized in the left temporal areas for δ-γ_1_ and θ-γ_1_ and in the left parietal areas for θ-β (Figure 2B, lower part).

### Differences on network properties

**Figure 3:**
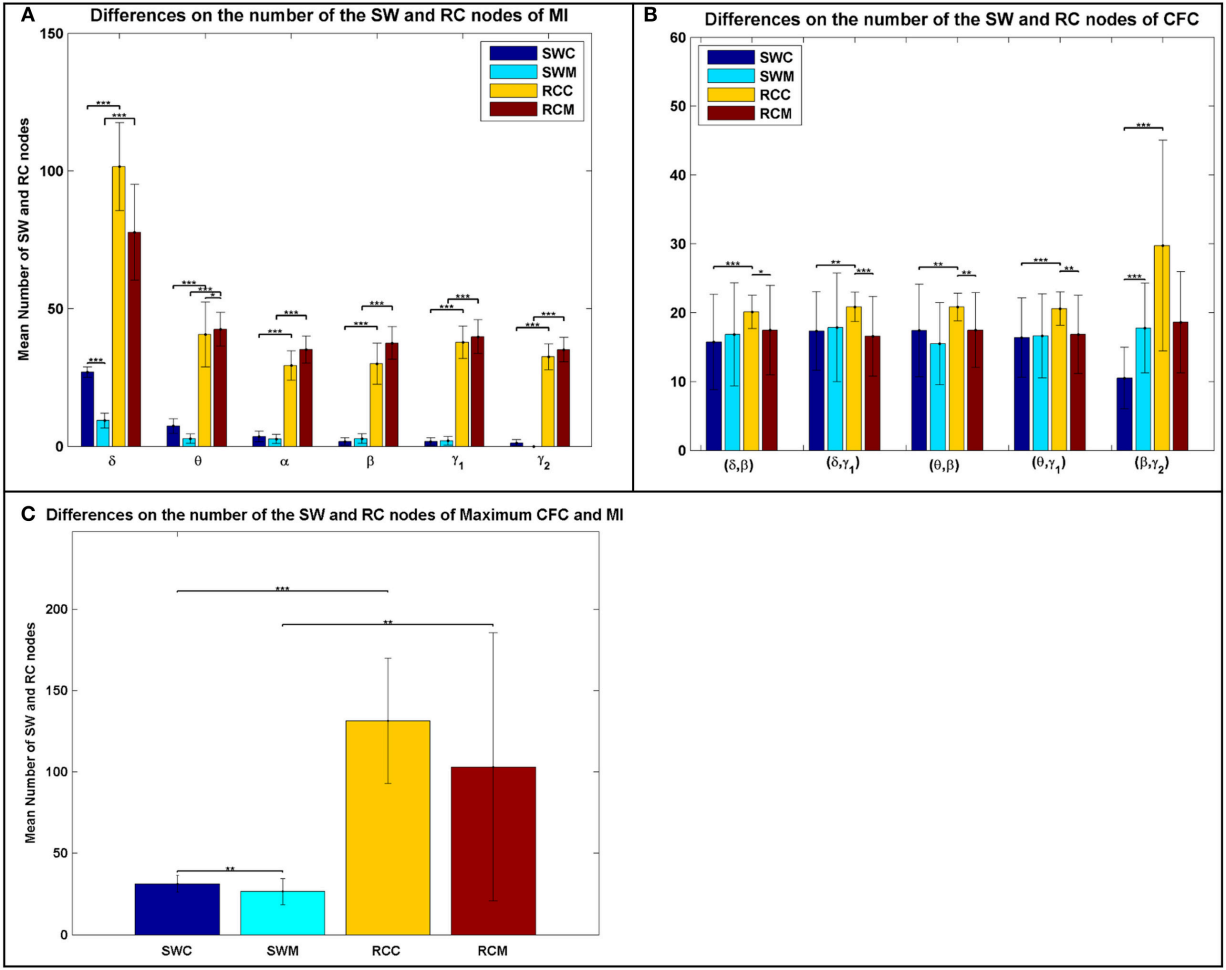
Average number of SW and RC nodes for **(A)** IFCG; **(B)** CFCG, and **(C)** ICFCG. Statistical comparisons between SW and RC nodes for each group (SWC: SW Control, RCC: RC Control, SWM: SW mTBI, and RCM: RC mTBI) and between groups for each network organization (SWC vs. SWM and RCC vs. RCM). All comparisons (paired test linked by ^*^) reach statistical significance (*p*-value: ^*^ < 0.05; ^**^ < 0.01, and ^***^ < 0.001).

In addition to the average distributions, the average number of the RC nodes was significantly higher than the SW nodes (Figure 3), indicating a stronger DP for the RC organization, for each type of FCG. Following the statistical analysis presented in the main text and elsewhere (Antonakakis et al., 2015, 2016), we investigated whether, and under what conditions, the number of SW and RC nodes were significantly different. Figure 3 illustrates the average number of nodes for both network organizations and all types of FCGs, groups, frequency bands, and frequency pairs. Most cases showed a significantly higher number of RC nodes than SW. Moreover, a significantly higher number of SW nodes existed for the control group in most frequency bands, but the number of RC nodes for controls was only higher in the δ band for IFCGs (Figure 3A). In all other cases, the number of both SW and RC nodes was significantly higher in the mTBI group compared to the control group. For CFCGs (Figure 3B), statistical differences were observed between the number of SW and RC nodes in the mTBI group for frequency pairs (δ, β), (δ, γ_1_), (θ, β), and (θ, γ_1_), but in the case of controls, significant differences were observed in all frequency pairs. However, the number of RC nodes was significantly higher in the control group compared to mTBI. Finally, in the case of ICFCGs (Figure 3C), the number of hubs in both network organizations was different between and within the two groups, with the number of RC nodes and their differences across groups being higher than the corresponding SW values.

**Figure 4:**
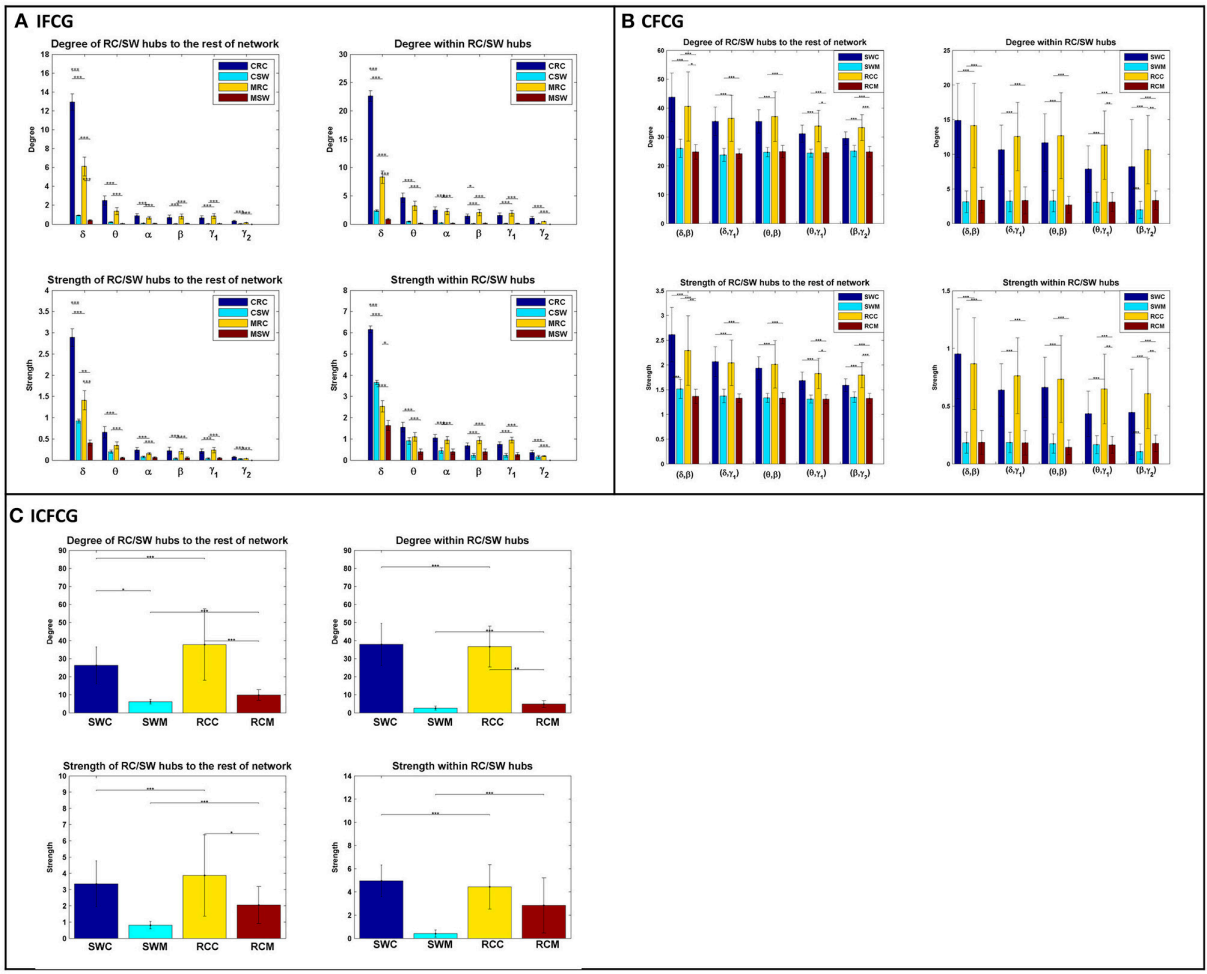
Representation of the averaged degree and strength of the RC/SW hubs (i) with the rest of nodes networks and (ii) within RC/SW nodes across each group (acronyms follow the definition in capture of Figure 3). For **(A)** IFCG, **(B)** CFCG, and **(C)** ICFCG. All comparisons (paired test linked by ^*^) reach statistical significance (*p*-value: ^*^ < 0.05; ^**^ < 0.01, and ^***^ < 0.001).

The bar graphs in Figure 4 show the average degree and strength values of RC/SW subnetworks in two connectivity configurations: (a) connections of RC/SW hubs to the rest of the network nodes and (b) connections within the RC/SW structure. The above analysis was performed for every FCG type and every frequency band or pair of frequency bands. In the case of IFCGs (Figure 4A), both the degree and strength of RC were significantly higher than the corresponding SW values in both groups, in all bands, and all types of network (RC/SW). In addition, both network properties were higher in the control group compared to the mTBI group in both types of network (RC/SW). In particular, the mean value of degree and strength were significantly higher in the control group compared to mTBI in the RC topology and the δ band.

**Figure 5:**
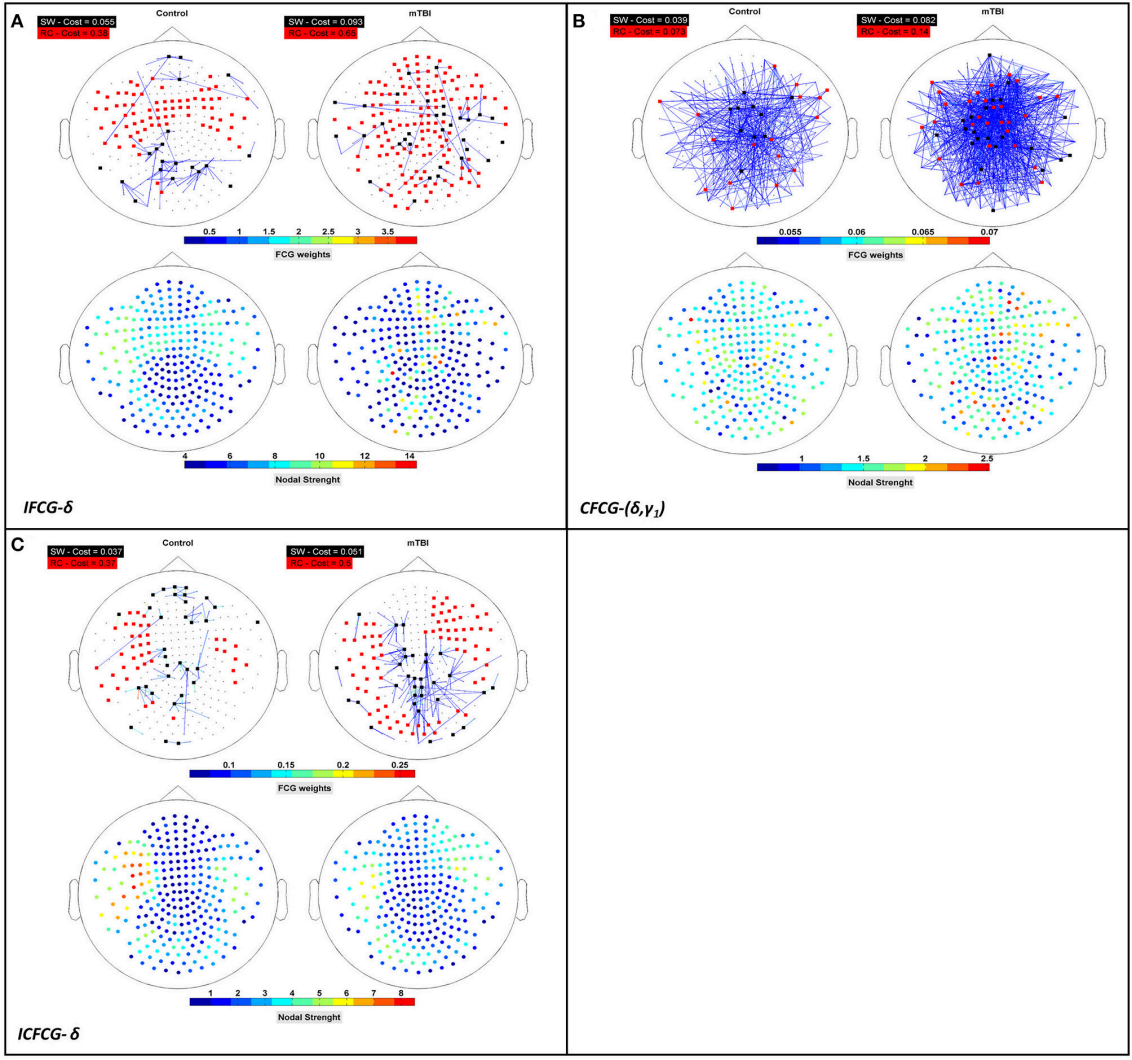
Representation of the SW network (colored edged + black-washed SW nodes on top topographies for each sub-image) simultaneously with the RC topology (red nodes on top topographies for each sub-image) and the corresponding nodal strengths (colored bottom topographies for each sub-image) for each FCG type **(A)** IFCG **(B)** CFCG, and **(C)** ICFCG in specific frequency bands and frequency pairs. Each color bar is common to the control and mTBI groups.

Regarding CFCGs (Figure 4B), the average values of degree and strength in RC were higher than in SW, for both groups. Apart from the first frequency pair (δ, β), significant differences were observed between the two groups in both network topologies, in terms of network metrics (strength, degree) and connectivity for the rest of frequency pairs. In both the CFCG and ICFCG cases, statistical differences are found in both groups between the RC and SW topologies in terms of degree and strength (Figure 4C). However, no statistically significant differences were detected between the groups in both network metrics and conditions for the SW topology (Figure 5C).

Overall, the SW organization showed stronger activation mostly in frontal and central regions for the normal control group in all frequency bands except for the beta band (Figure 2). Similarly, the RC organization showed stronger activations in left temporal areas for all frequency pairs examined {fc = (fl,fh) = [(δ,β), (δ,γ1), (θ,β), (θ,γ1) and (β,γ2)]}. However, in the mTBI group activation was stronger in right frontal and temporal areas (Figure 2). Finally, the control group showed significantly higher degree and strength than the mTBI group in all frequency bands and frequency pairs for each FCG type (Figure 4).

### The relation between rich-club and small-world organizations

To verify that RC network organization presents complementary information to the SW form, we superimposed both structures on the upper parts of Figures 5A–C, for three cost values, respectively. The SW network organization is shown with black nodes connected by the outflow edges, while the RC network organization is shown with red nodes, proving that RC nodes can be found within an SW network. The nodal strength of each FCG type is illustrated in the lower part of Figures 5A–C, to show the hub nodes in specific frequency bands and pairs of frequencies. The first observation associated with Figure 5 is that most RC nodes are part of the SW network, since in each sub-image some nodes belong simultaneously to both organizations, demonstrating that a node can be simultaneously an SW and an RC node. Furthermore, the spatial distribution of the upper and lower topography in each figure indicates that the RC nodes appear to have higher nodal strength than the SW nodes (upper and lower Figures 5A–C) for all cases. Therefore, the RC network organization reveals a higher information flow within an FCG.

## Results of network attacks on brain models

### Attacks on intra-FCG brain models

**Figure 6:**
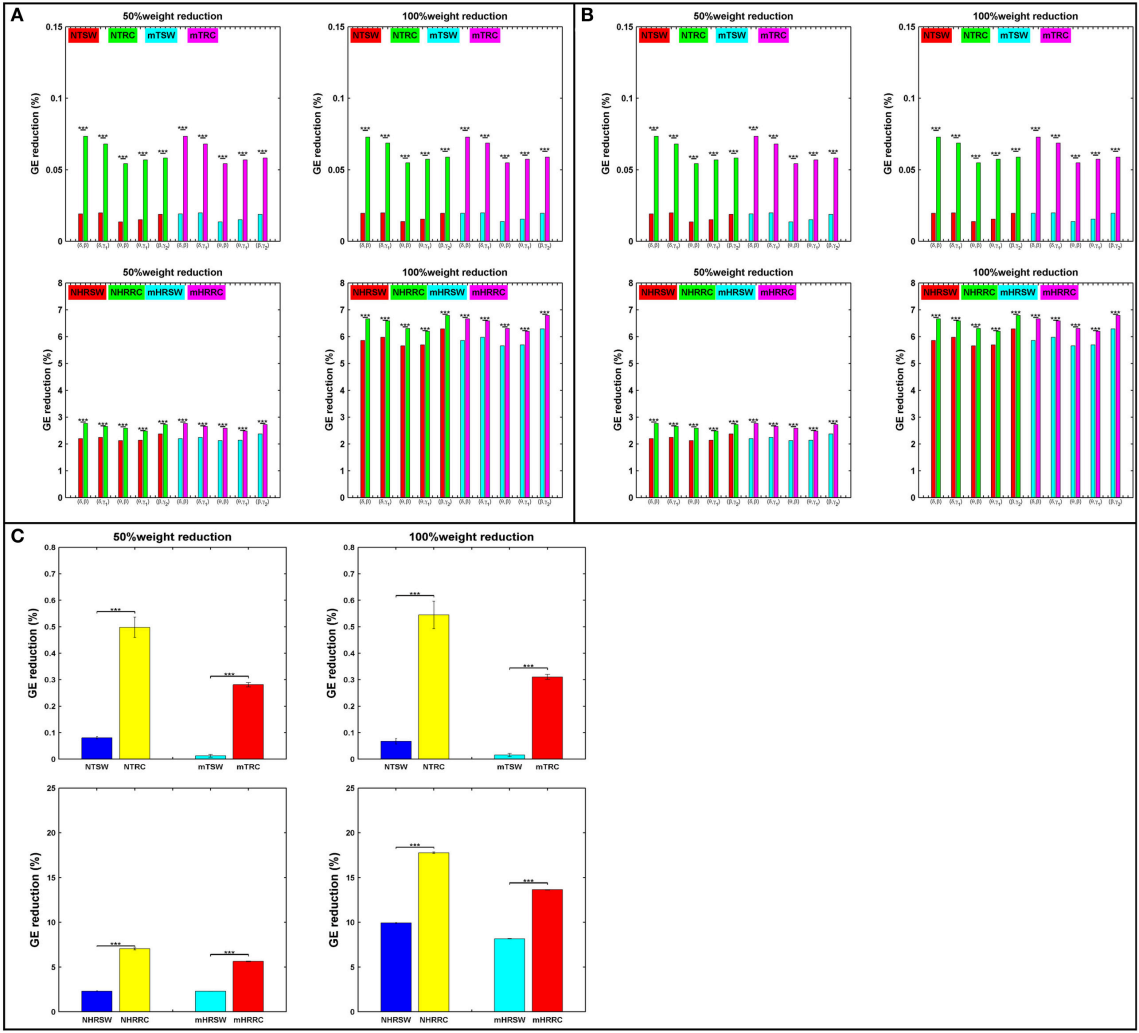
Percent reduction in total GE on SW and RC brain models for **(A)** IFCG, **(B)** CFCG, and **(C)** ICFCG after attacking targeted interconnected nodes (T) or hubs connected to random nodes (HR). Control: NTSW and NHRSWred; NTRC and NHRRCgreen; mTBI: mTSW and mHRSWcyan; mTRC and mHRRCmagenta. In case of **(C)** the color-bars are different, Control: NTSW and NHRSWblue and NTRC and NHRRCyellow; mTBI: mTSW and mHRSWcyan and mTRC and mHRRCred. The scale in **(A,B)** is the same. Network attack: select 50% of connections that intra-connect hubs within either RC or SW backbone or 50% of connections that inter-connect hubs with the rest of the network. All comparisons reached statistical significance (*p*-value: ^***^ < 0.001).

Attack strategies on the RC and SW organizations are presented in terms of percentage of GE reduction as shown in Figure 6. For each iteration, we randomly chose 50% of the links within the SW or RC nodes (TSW and TRC), or the interconnections between RC/SW nodes and the rest of the network (HRSW and HRRC). Statistical analysis were performed between SW and RC network topologies in all the aforementioned scenarios. In most cases, the RC organization showed significantly higher %GE reduction. After each attack, both the SW and RC organizations changed compared to their initial structure.

First, the average %GE reduction of TRC and HRRC for all cases [NTRC – mTRC and NHRRC – mHRRC, (here the prefixes “N” and “m” denote the normal control and mTBI groups, respectively)] and across all iterations was significantly higher (*p*-value < 0.001) compared to the TSW and HRSW, with the exception of the γ_2_ band for controls and the α band for mTBI patients, in the case of IFCGs (Figure 6A). The %GE reduction did not exceed 4% in any case for the target attack, but in the case of the hub, the rest of the network attacks demonstrated a much larger damage in the network integration with levels close to 20% decrease for 50% of weight reduction, and 45% decrease for 100% weight reduction (Figure 6A).

Regarding the %GE reduction, the results based on the CFCGs were similar to those based on IFCGs. In particular, it can be seen (Figure 6B) that the RC organization showed a statistically significant higher %GE reduction than the SW organization. Overall, the %GE reduction was no more than 7% in all cases. Finally, the %GE reduction in the case of mFCGs (Figure 6C) also showed significantly higher %GE for the RC organization than the SW.

### Synchronization within the RC subnetwork

**Figure 7:**
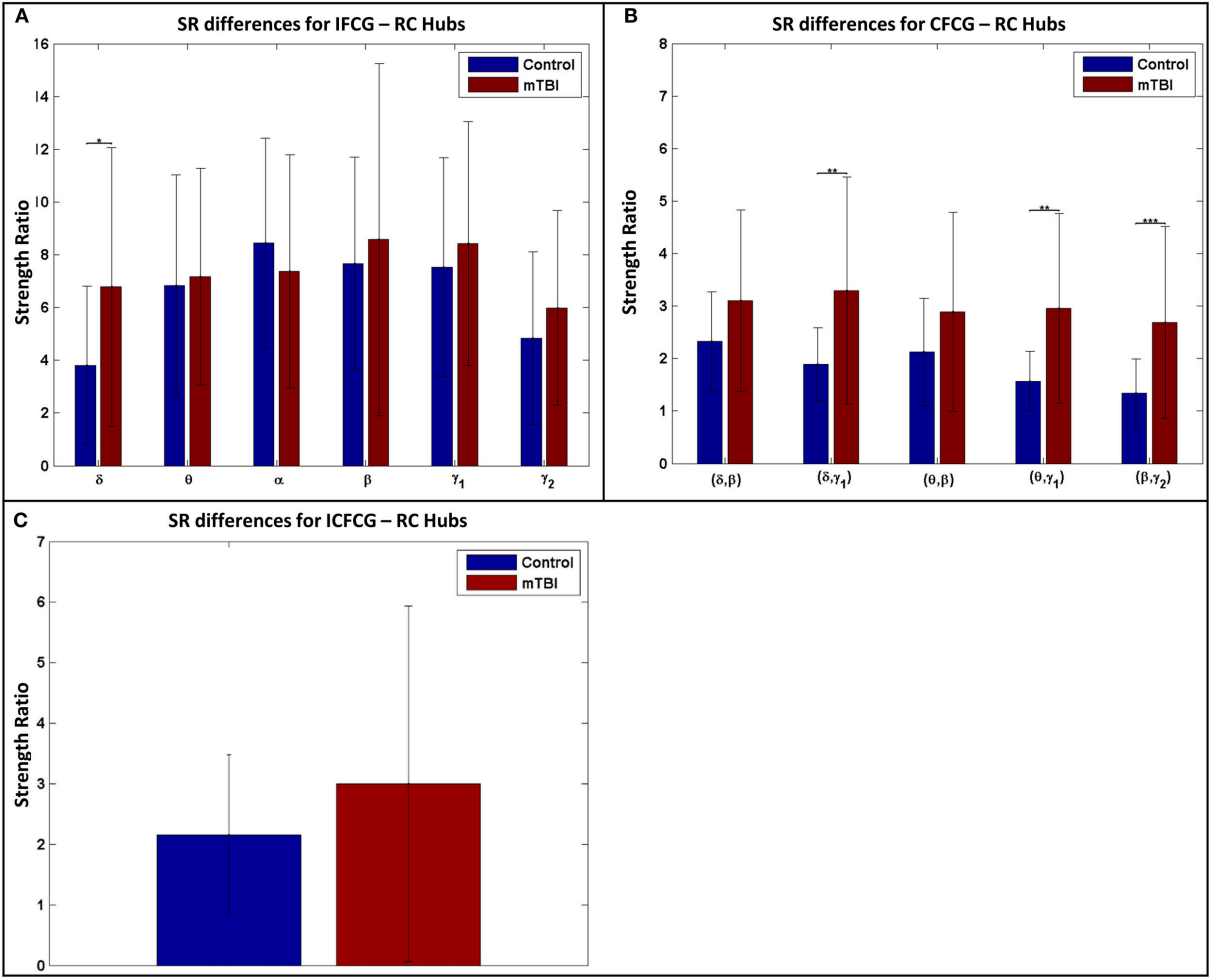
Strength ratio SR for **(A)** IFCGs, **(B)** CFCGs, and **(C)** ICFCGs. All comparisons (paired test linked by ^*^) reached statistical significance (*p*-value: ^*^ < 0.05; ^**^ < 0.01, and ^***^ < 0.001).

The results of the strength ratio SR are presented in Figure 7 for each type of FCG. All cases demonstrated a higher ratio for the mTBI group, except in the α band for the IFCG-RCs (Figure 7A). In addition, statistical analysis performed to detect possible differences between the control and mTBI groups found significant differences in the δ band in the IFCG-RC case (Figure 7A) and the frequency pairs (δ, γ_1_), (θ, γ_1_), and (β, γ_2_) in the CFCG-RC (Figure 7B) and ICFCG-RC (Figure 4C) cases. Overall, the RC hub nodes presented higher SR values than the simple nodes as seen in Figure 7. Additional results, especially for the RC hubs, are presented in the Supplementary Material.

**Figure 8:**
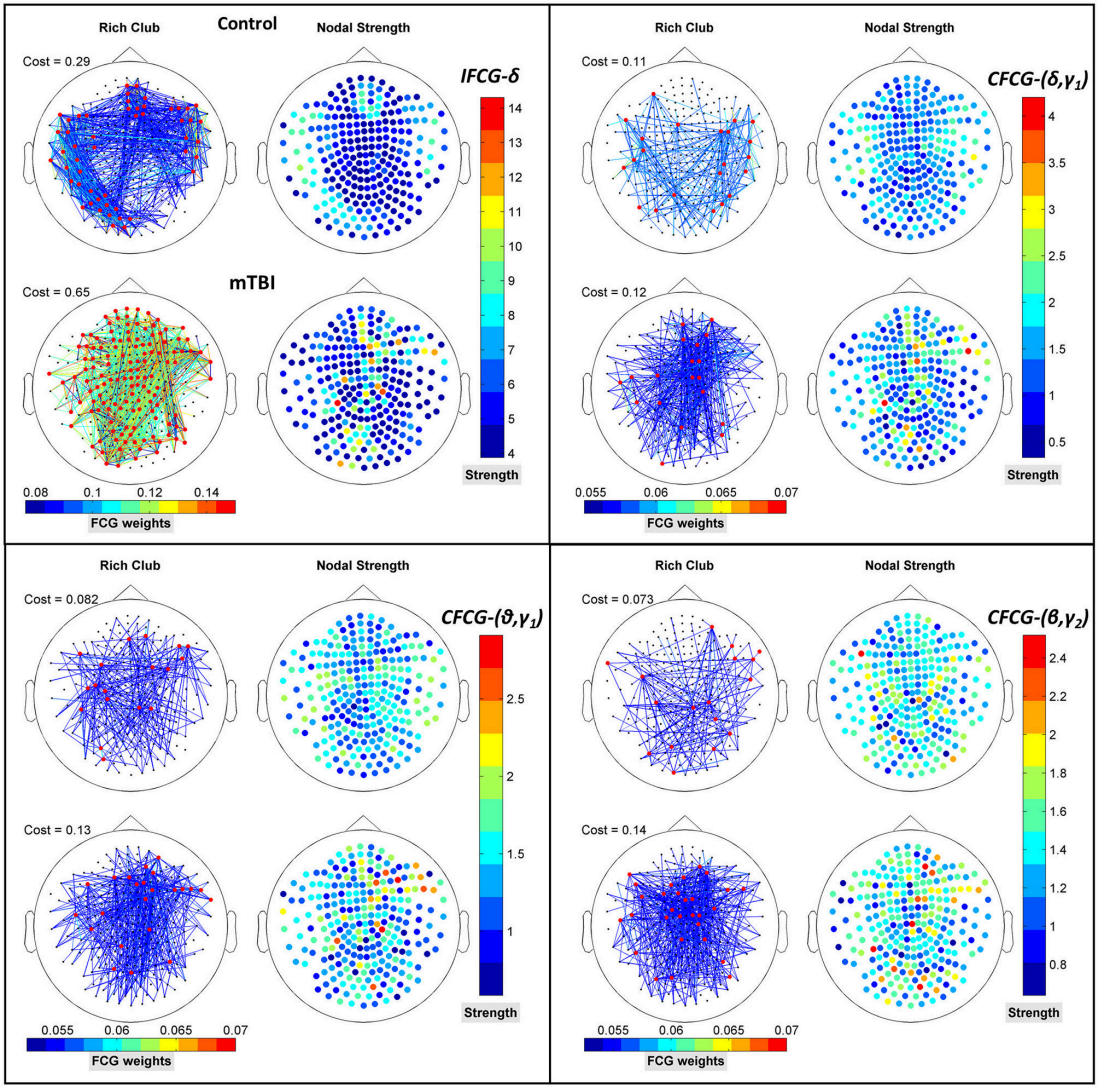
Representation of the RC topology (red nodes on left topography for each sub-image) for each statistically significant case of Figure 7 with the nodal strength (colored right topography for each sub-image) of the corresponding FCG and edge cost. Each color bar is common to the control and mTBI groups.

Figure 8 further illustrates the spatial distribution of connections from RC hubs (Figure 8: red points) to the rest of the nodes (Figure 8: black nodes) along with the strength of the nodes for each type of FCG. In particular, the edge-cost (Figure 8: cost), which is the ratio of the total strength of RC nodes to the total strength all of nodes of the corresponding full weighted unthresholded FCGs, is higher for mTBI patients compared to control subjects. In this case, the number of the mTBI RC edges is significantly higher from normal control RC edges.

In addition to the above results, an prominent relation was revealed for the differences within each brain area between the two groups. Namely, higher (mTBIs > Controls) and significant (*p*-value < 0.05) mean probability values existed in the left parietal lobe in the δ and β bands, right frontal for the (θ-γ1) frequency pair in the case of IFCG, and in left occipital areas for the (θ-β) frequency pair of the CFCG. Additional significant differences (*p*-value < 0.05, Controls > mTBIs) were seen in left temporal (δ band of IFCGs), left occipital (δ-β, δ-γ1, θ-β and β-γ2 of CFCG), left temporal (δ-γ1 and θ-γ1 of CFCGs), and right parietal (β-γ2 of CFCGs) areas.

## Discussion

In the present study, we compared the RC and SW organizations under three different FCG representations, namely IFCG, CFCG, and ICFCG. Our aim has been to develop reliable biomarkers for the accurate detection of network abnormalities caused by mTBI by analyzing functional brain connectivity profiles that combine the RC organization with prominent MEG intrinsic coupling modes. We assumed that the highest reduction in %GE would reveal the organization that best described the topology of the “true” underlying brain networks. Our results showed that separation of the mTBI patients from the normal control group was feasible with ~91% accuracy based on the ICFCG representation (Table 1).

The importance of the RC organization was first investigated in neuroimaging brain studies (Van den Heuvel and Sporns, 2011) using diffusion tensor imaging (DTI) that emphasized the role of RC organization in information integration and in conferring robustness to its structural core. Since then, many human connectomic studies have followed (Van den Heuvel et al., 2013; Senden et al., 2014; Vértes and Bullmore, 2015; Crossley et al., 2016) mostly using DTI or fMRI on patients and normal control subjects. Recently, we investigated a prominent RC organization obtained from MEG recordings of spontaneous brain activity of mTBI patients for a specific cross-frequency pair (Antonakakis et al., 2015). In another recent study, we presented a promising imaging method for detecting network abnormalities caused by mTBI during task-free spontaneous MEG activity based on intra- (Dimitriadis et al., 2015b) and cross-frequency coupling (Antonakakis et al., 2015, 2016). In this study, we performed a thorough investigation on these issues based on different interaction metrics, network formation approaches, and evaluation scenarios, over all frequency bands and band pairs. We observed a significant direct dominance (Figure 2) of low frequency bands and band pairs regarding the distribution of RC nodes (Figure 2) for both groups. More specifically, temporal areas played a crucial role in the definition of the CFCG-RC topology in both groups. The RC nodes for the mTBI group were mainly seen in right temporal areas compared to controls (Figure 2B).

In a previous analysis of the same dataset, we classified correctly the two groups with more than 90% accuracy (Antonakakis et al., 2015, 2016) using only the CFCG frequency pairs. A recent study using resting state MEG (Dunkley et al., 2015) showed slow abnormal brain activity in mTBI patients. The trend was an increase of low frequency amplitude in patients with mTBI, and this spectral alteration appeared most prominently in temporal regions. In numerous studies, these findings have been linked to brain injury (Lewine et al., 2007; Huang et al., 2014). We found reliable evidence (Figure 2) that the highest SW and RC distributions were mainly located over temporal areas. The absence of significant differences in the distribution of the CFCG-SW (Figure 2B, upper part) was also reported by a recent study (Athanasiou et al., in press) using the complementary modalities of EEG/MEG and multivariate functional connectivity. Over-representation in right frontal areas has also been found by previous studies, suggesting that the effects of mTBI are more prevalent in frontal regions which are more vulnerable to brain injury (Eierud et al., 2014; Antonakakis et al., 2016). A recent MEG study with a mixed group of mild, moderate, and severe TBI patients showed reduced FC in frontal areas bilaterally and increased FC in left temporo-parieto-occipital regions and in the right thalamus (Tarapore et al., 2013).

Revealing a consistent over-representation of hubs in right frontal areas in mTBI subjects across all frequency bands provides further support that structural alterations cause these frequency changes over the whole frequency spectrum (Dunkley et al., 2015). The significant over-representation of hubs in left occipital brain areas (Figure 2B, lower part) of the mTBI group may be indicative that, unconsciously, subjects were experiencing mental images caused by the trauma as spontaneous brain activity. It would be interesting to explore the activity of mTBI patients in the fusiform gyrus and compare it to controls using neuromagnetic source reconstruction (Zhan et al., 2016). Further analysis on the source level could reveal if there is increased connectivity between visual system and default mode network.

Furthermore, we found that, compared to SW, the RC organization underwent significantly higher GE changes in both groups, with a much larger reduction in the mTBI group. These findings indicate that the RC organization can encode important topological features of the underlying brain networks (Figure 6). These findings in particular extend our previous results from comparing the SW and RC models only on CFCGs and in the δ-β frequency pair (Antonakakis et al., 2015). Through the adopted attack strategy, the RC organization demonstrated higher levels of %GE reduction and confirmed the damage inflicted on a node by attack, which was simulated as a decrease in the weights of the node's connections (Van den Heuvel and Sporns, 2011). Mišić et al. (2014) also demonstrated that a large part of information flows in the RC organization, suggesting that it is better modeled by an RC organization than an SW. The reduction of GE in mTBI could be linked to the significant reduction of functional connectivity within the default mode network revealed by a recent mTBI study working with MEG resting-state on the source level (Alhourani et al., 2016). In a next study working at the source level, we will try to reproduce our results.

Additionally, to uncover the different role and definition of hubs in a brain network, we explored the mean degree and mean strength of RC and SW hubs in two conditions, namely within their subnetworks and with the rest of the network. Our analysis revealed significantly higher mean values in degree and strength in the control group compared to the mTBI in RC topology for both conditions in the δ and θ bands (Figure 4A). Based on these findings, we conclude that the RC definition can uncover the subset of nodes from a network that plays a pivotal role in global information integration. A collapse of such highly interconnected hub regions can cause communication aberrations between different parts of the brain (Van den Heuvel and Sporns, 2011). Thus, these brain hubs should be further studied both structurally and functionally in various brain diseases and disorders.

Through the examination of the strength ratio of RC nodes (Figure 7), we found hyper synchronization in mTBI patients compared to normal controls within RC subnetworks based on intra- and inter-frequency intrinsic couplings in the δ, δ-γ_1_, θ-γ_1_, and β-γ_2_ frequencies and frequency pairs. These results agree with the basic findings of Hillary et al. (2014) who showed that brain connectivity increased after TBI— they examined the mean degree of 52 nodes and showed significantly greater connectivity in TBI patients compared to normal controls. Instead, the highest degree nodes (i.e., RC nodes in our case) were selectively observed in several core subnetworks (either TRC or HRRC). Our analysis further explains hyper-connectivity in mTBI patients under the RC model and the use of both intra- and inter-frequency coupling.

In conclusion, this study demonstrated that the RC organization of graphs reflecting the local distribution of activity from resting-state brain networks can encode characteristic aspects of two types of FCG for both mTBI patients and control subjects. RC hubs in mTBI subjects were overrepresented in right temporal areas from θ to γ_1_ frequencies and underrepresented in left occipital areas in δ-β, δ-γ_1_, θ-β, and β-γ_2_ frequency pairs. Therefore, our analysis does not only support the use of resting state MEG for the extraction of meaningful features that describe abnormal brain connectivity after mTBI (Buzsáki and Watson, 2012; Zouridakis et al., 2012; Huang et al., 2014; Dimitriadis et al., 2015b; Dunkley et al., 2015; Antonakakis et al., 2016) it also indicates the need to explore the RC organization under different types of interactions (intra- and cross-frequency) for the development of complementary connectomic biomarkers of recovery from mTBI that can be useful in clinical research (Buzsáki and Watson, 2012). The potential functional implications of RC organization of MEG intrinsic coupling modes, considering its role in network integration and its vulnerability in various disorders like mTBI, seem to deserve further investigation for diagnostic and clinical purposes. Furthermore, our approach is suitable for accessing the recovery process following mTBI using resting state MEG (Zouridakis et al., 2016; Li et al., 2017) and focusing not only on the strength of the couplings but also on the dominant type of interactions (Bharath et al., 2015; Losoi et al., 2015).

In order to provide a robust spatial mapping for brain functions in both control subjects and mTBI patients, it is necessary to adopt a dynamic functional connectivity approach (Dimitriadis et al., 2012, 2013a, 2014, 2015a,c, 2016) through the definition of functional connectivity microstates (Dimitriadis et al., 2013a) and/or network microstates (Dimitriadis et al., 2015c). Our current studies focus on exploring a dynamic combination of IFCGs and CFCGs into ICFCGs (Dimitriadis et al., 2016), their related microstates, and their symbolic dynamical signature in a possible combination with compression methods (Luo et al., 2013).

## Author contributions

MA is currently a Ph.D. student in the Institute of Biomagnetism and Biosignal analysis. MA has performed the whole data analysis and written the first draft of manuscript. SD significantly contributed his ideas for the structure of the pipeline and co-supervised together with MZ the analysis of the data. AP offered the data-set and his old group was responsible for the collection of it. GZ developed with his experience the main body of the manuscript and made the final check of the whole manuscript.

### Conflict of interest statement

The authors declare that the research was conducted in the absence of any commercial or financial relationships that could be construed as a potential conflict of interest.

## Footnotes

^1^http://www.cad.zju.edu.cn/home/dengcai/Data/DimensionReduction.html
