# Supplementary material for: Altered Rich-Club and Frequency-Dependent Subnetwork Organization in Mild Traumatic Brain Injury: A MEG Resting-State Study
Source: Front Hum Neurosci. 2017 Aug 30;11:416. doi: 10.3389/fnhum.2017.00416 (PMC5582079; doi:10.3389/fnhum.2017.00416)
Supplement: Supplementary file 1 [file DataSheet1.docx]

Supplementary Material

Altered Rich Club and Frequency-Dependent Subnetworks Organization in Mild Traumatic Brain Injury: A MEG Resting-State Study

Marios Antonakakis^1, 2, *^, Stavros I. Dimitriadis^3-6^, Michalis Zervakis^2^, Andrew C. Papanicolaou^7^, and George Zouridakis^8^

1. Institute of Biomagnetism and Biosignal Analysis, Westfalian Wilhelms-University Muenster, Muenster 48149, Germany, email: [marios.antonakakis@uni-muenster.de](mailto:marios.antonakakis@uni-muenster.de)
2. Digital Image and Signal Processing Laboratory, School of Electronic and Computer Engineering, Technical University of Crete, Chania, 73100 Greece
3. Institute of Psychological Medicine and Clinical Neurosciences, Cardiff University School of Medicine, Cardiff, United Kingdom
4. Cardiff University Brain Research Imaging Center (CUBRIC), School of Psychology, Cardiff University, Cardiff, United Kingdom
5. Artificial Intelligence and Information Analysis Laboratory, Department of Informatics, Aristotle University, Thessaloniki, 54124, Greece
6. [Neuroinformatics.GRoup](http://neuroinformatics.gr), Department of Informatics, Aristotle University, Thessaloniki, Greece
7. Departments of Pediatrics, and Anatomy and Neurobiology, University of Tennessee Health Science Center, and Neuroscience Institute, Le Bonheur Children's Hospital, Memphis, TN, USA
8. Biomedical Imaging Lab, Departments of Engineering Technology, Computer Science, Biomedical Engineering, and Electrical and Computer Engineering, University of Houston, Houston, TX 77204, USA

*Corresponding author: Marios Antonakakis, phone: +49 2518352547, +30 2821037206, Fax: +30 2821037542, email: [marios.antonakakis@uni-muenster.de](mailto:marios.antonakakis@uni-muenster.de).

1. **Data Preprocessing**

Preprocessing of the MEG recordings was performed using Matlab (The MathWorks, Inc., Natick, MA, USA) and Fieldtrip (Oostenveld et al., 2011). First, activity on bad MEG channels was replaced using interpolation (Oostenveld et al., 2011) on the four closest channels surrounding the bad one. Then, a notch filter reduced the effects of line noise at 60 Hz and it was followed by independent component analysis to separate cerebral from noncerebral activity (Delorme and Makeig, 2004). Subsequently, the data were whitened and reduced in dimensionality using principal component analysis with a threshold set to 95% of the total variance (Delorme and Makeig, 2004; Escudero et al., 2011; Antonakakis et al., 2013, 2015, 2016). The statistical values of kurtosis, Rényi entropy, and skewness of each independent component were used to eliminate ocular and cardiac artifacts. Specifically, a component was deemed artifactual if more than 20% of its values after normalization to zero-mean and unit-variance were outside the range of [-2, +2] (Delorme and Makeig, 2004; Escudero et al., 2011; Antonakakis et al., 2013, 2015, 2016). Data from all subjects were used in the analysis presented below.

1. **Estimation of Phase-to-Amplitude Coupling - PAC**

Given a multidimensional array of time series **X,** PAC was calculated for the data from each sensor X­_i_ and between pairs of sensors Χ_i_ and X_j_, with i, j = 1 … 248, using mutual information (MI) (Tsiaras et al., 2011; Bullmore et al., 2011). First, we extracted the low-frequency phase (f_l,i_) of the i-th component $\varphi_{f_{l},i}$ and the high-frequency amplitude (f_h,j_) of the j-th component A_fh,j_ using the Hilbert transformation (HT) (Claerbout, 1985). More specifically, f_h_ covered frequencies from θ to γ_2,_ whereas f_l_ varied from δ to γ_1_. The cutoff frequency of the lowpass filter was higher than the cutoff of the highpass, so that the two filtering operators preserved a common bandpass interval. Since the power spectrum of A_fh,j_ preserved only a small portion of the very high frequencies, we bandpass filtered it to match the frequency range of $\varphi_{f_{l},i}$. Then, the phase of A_fh,j_, denoted by $\varphi_{f_{h},j}^{f_{l},i}$, was extracted by a second HT. Finally, the estimation of PAC*_fc_* was performed through Eq. (1), where ***Z =*** $\varphi_{f_{l},i}$, ***Y=*** $\varphi_{f_{h},j}^{f_{l},i}$, and *f_c_* = (*f_l_,f_h_*) = [(δ,θ), …, (γ_1_, γ_2_)].

To compute the Cross Frequency Functional Connectivity Graphs (CFCG) - PAC values, we used the HT to estimate the phase ($\varphi_{f,i}$) and amplitude ($A_{f,i}$) of every $X_{f,i}$, separately in each frequency band using

$$\varphi_{f,i}=\tan^{-1} \left( \frac{Im\left( HT\left( X_{f,i} \right) \right)}{Re\left( HT\left( X_{f,i} \right) \right)} \right) (1)$$

and

$$A_{f,i}=\left| \sqrt{Im\left( HT(X_{f,i}) \right)^{2}+Re\left( HT(X_{f,i}) \right)^{2}} \right| (2)$$

where $Im(HT(X_{f,i}))$ $Im(HT(X_{f,i}))$ and $\mathrm{Re}\left( HT(X_{f,i}) \right)$ $\mathrm{Re}\left( HT(X_{f,i}) \right)$ are the imaginary and real parts of $HT(X_{f,i})$ $HT(X_{f,i})$, respectively. We then applied a band-pass filter to $A_{f,i}$ $A_{f,i}$ using the same filter parameters used to extract $X_{fl,i}$ $X_{fl,i}$, which resulted in a new time series, $A_{fh,fl,i}$ $A_{fh,fl,i}$. A second HT was then used to extract the phases of the $f_{l}$ $f_{l}$-filtered $f_{h(high)}$ $f_{h(high)}$ amplitude envelope $(\varphi_{fh,fl,i}$ $(\varphi_{fh,fl,i}$) (Voytek et al., 2010). The estimation of PAC between the phase of low frequency $f_{l}$ $f_{l}$,$\varphi_{f_{l},i} ,$ $\varphi_{f_{l},i}$and the amplitude of the high frequency$f_{h},$ $f_{h},$ $\varphi_{{fh,f}_{l},i} ,$ $\varphi_{{fh,f}_{l},i}$ between two sensors $X_{i}\mathrm{and}X_{j}$ $X_{i}\mathrm{and}X_{j}$, is given by Eq. (1) in the main text, where $X=\varphi_{f_{l},i}\mathrm{and}Y=\varphi_{{fh,f}_{l},i}$ $Z=\varphi_{f_{l},i}\mathrm{and}Y=\varphi_{{fh,f}_{l},i}$.

1. **Surrogate Data Analysis**
   1. **Intra and Inter-Frequency Coupling**

To identify significant intra- and cross-frequency interactions for every frequency and pair of frequencies within and between all 248 sensors, we employed surrogate data analysis (Theiler et al., 1992) to determined (a) if a given PAC value differed from what would be expected by chance alone, and (b) if a given non-zero PAC indicated coupling that was statistically non-spurious.

For every sensor pair, frequency, and pair of frequencies, we tested the null hypothesis H_0_ that the observed PAC value came from the same distribution as the distribution of surrogate PAC values. One thousand surrogate time series *ϕsLF*(*t*) were generated by cutting at single point at a random location and exchanging the two resulting time courses (Canolty et al., 2006; Aru et al., 2015). Repeating this procedure produced a set of surrogates with minimal distortion of the original phase dynamics and impact on the nonstationarity of the brain activity compared to either shuffling the time series or cutting and rebuilding the time series in more than one time point. This procedure ensures that the observed and surrogate indices shared the same statistical properties. For each dataset, the surrogate MI (MI^s^) was computed. We then determined a one-sided *p*-value expressing the likelihood that the observed PAC value could belong to the surrogate distribution and correspond to the proportion of “surrogate”' MI^s^ which was higher than the observed MI value (Theiler et al., 1992). MI values associated with statistically significant *p*-values were considered unlikely to reflect signals not entailing MI coupling.

The false discovery rate (FDR) method (Benjamini and Hochberg, 1995) was employed to control for multiple comparisons across all combinations of sensor pairs, independently for each frequency and frequency pair with the expected proportion of false positives set to *q* ≤ 0.01. Finally, only the significant connections were kept with their MI weights while the rest were substituted with zeros.

- 1. **Dominant Type of Intrinsic Coupling Mode**

The (Intra – Inter/Cross FCG) ICFCG with the dominant type of interaction were constructed as follows. The FDR method (Benjamini and Hochberg, 1995) was employed to control for multiple comparisons (across frequencies and all possible pairs of frequencies) with the expected proportion of false positives set to *q* ≤ 0.01. The MI mode that characterized a specific pair of frequencies was determined based on the highest statistically significant MI value from the surrogates. Finally, for each subject, we created two 2D matrices with dimensions 248x248, one for keeping the MI strength and a second one to code the dominant type of interaction {1 for δ, 2 for θ, … 21 for β-γ}.

1. **Data Driven FCG Filtering**

The aforementioned procedures resulted in a matrix of FCG values between the time series across all possible pairs of sensors that was modeled as a fully connected, weighted, symmetric, and directed or undirected FCG. Aiming at reducing the maximum number of possible directed connections N in the current FCG (k=248 and k^2^=61504), the FCG, matrices were filtered out so that the pattern with the most significant connections could emerge. A topological data driven thresholding scheme was performed based on graph theoretic aspects of global information among the sensor links (Dimitriadis et al., 2015b; Bassett et al., 2009) so as to maintain the most significant connectivity patterns. We applied thresholding on each type of FCG for all subjects in the control and mTBI groups. The specific steps of the filtering procedure and the online available implementation^[[1]](#footnote-1)^ have been already described and evaluated in recent studies (Dimitriadis et al., 2015b; Antonakakis et al., 2015, 2016). An example of this procedure is given in Figure S1.


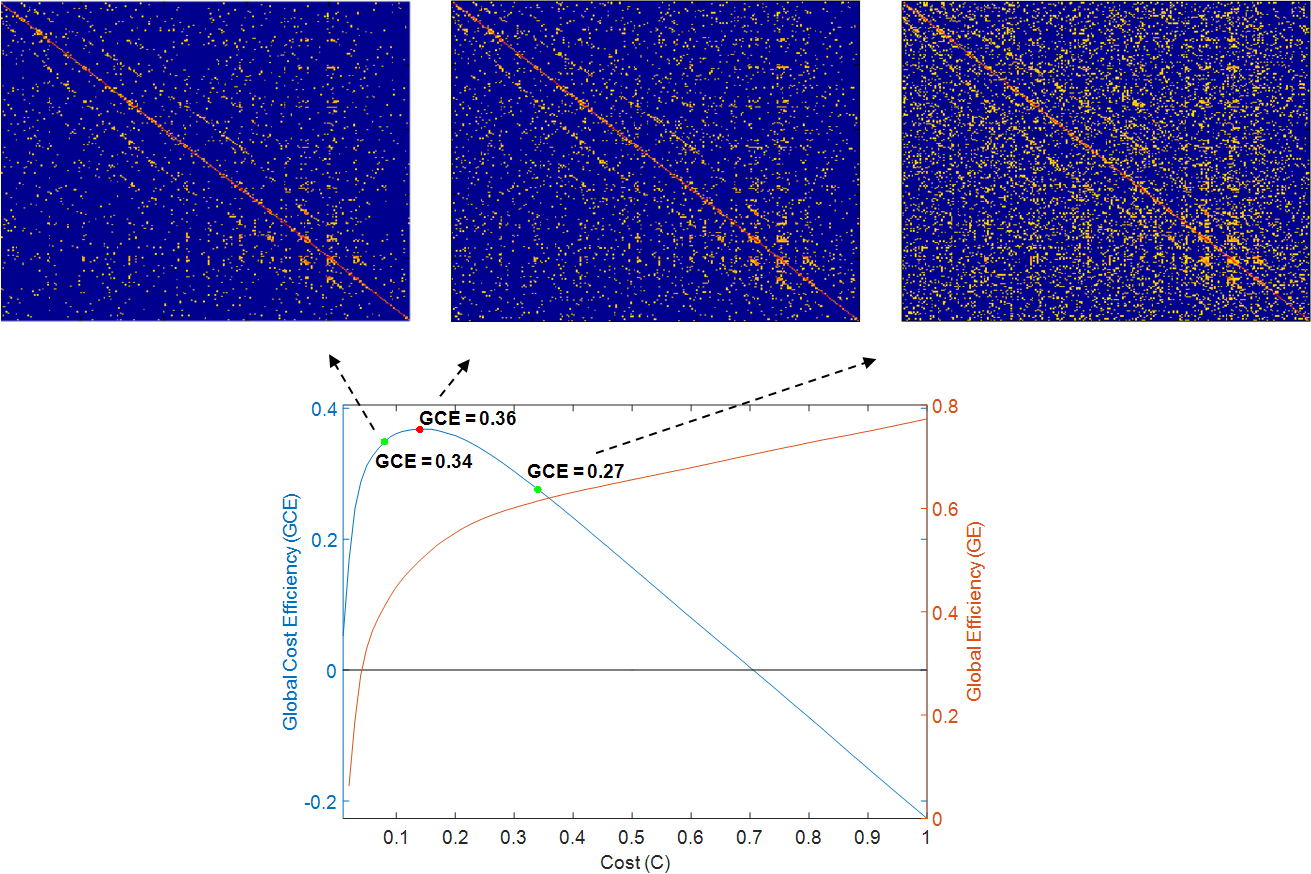


**Figure S1.** Global cost efficiency as a function of network cost. Three examples of graphs with significant links for the δ-β frequency pair from a control subject**.** The red dot corresponds to the maximum value (optimal threshold) of global cost efficiency while the green dots represent a non-optimal thresholds.

1. **Classification of Functional Connectivity Patterns**

The values of the IFCG, CFCG and ICFCG matrices are viewed as features in a high-dimensional space that can be used to classify the FCG obtained from individual subjects. In most studies, however, FCG are treated as vectors in a high-dimensional space (e.g., Shen et al., 2010; Pollolini et al., 2010; Richiardi et al., 2011), an approach that disregards the inherent tabular representation of FCG and their nature as second-order tensors. To overcome this limitation, we treat FCG as tensors and resort to tensor subspace analysis (TSA) for appropriate feature extraction (He and Cai, 2005). In our formulation, the tensor form was given as (subjects x sensors x sensors) (Dimitriadis et al., 2013a; 2015).

The TSA procedure blends multilinear algebra and manifold data learning. Given some FCG sampled from the space of functional connectivity patterns, the TSA approximation is modeled by first building an adjacency graph capturing the proximity relationships among the connectivity patterns and then deriving a tensor subspace that faithfully represents these relationships. TSA provides an optimal linear approximation to the FCG manifold.

Classification of FCG from individual subjects starts by computing the TSA representation and is followed by comparison with FCG of known labels. In our study, we used the k-nearest neighbor (kNN) algorithm (Duda et al., 2012) and the Frobenius norm (Horn and Johnson, 1990) as a measure of similarity. Apart from this classification scheme, indicated as “TSA+kNN”, we also employed TSA with ensemble classification (“TSA+ENS”), and TSA with extreme learning machine (ELM) classification (“TSA+ELM”).

To evaluate the performance of our strategy, a cross-validation scheme was followed. The entire set of individual FCG (control and mTBI) was randomly partitioned into two subsets, a *training set* (the database of FCG of known class or label) corresponding to 80% of the subjects (45 controls and 27 mTBI patients) and a *test set* (subjects for which the class had to be predicted) corresponding to the remaining 20% of the subjects (5 controls and 3 mTBI patients). As a measure of performance, we used the correct recognition rate (CC%) calculated as the proportion of subjects in the test set for which the correct label was predicted. The cross-validation scheme was repeated 100 times and the mean value and standard deviation of the overall performance, sensitivity, and specificity were estimated.

In addition, a multi-layer graph was created and validated using the same classification scheme for comparison with the above scheme. The multi-layer graph included the IFCG for every frequency band on the diagonal, and the CFCG for every frequency pairs at the corresponding frequencies, f_i_, f_j_, where i = δ, ..., γ­_1_ and j = θ, ..., γ­_2_. To better understand the structure of the multi-layer graph, Fig. S2 shows the mean value of the graph for the Normal Control and mTBI groups.


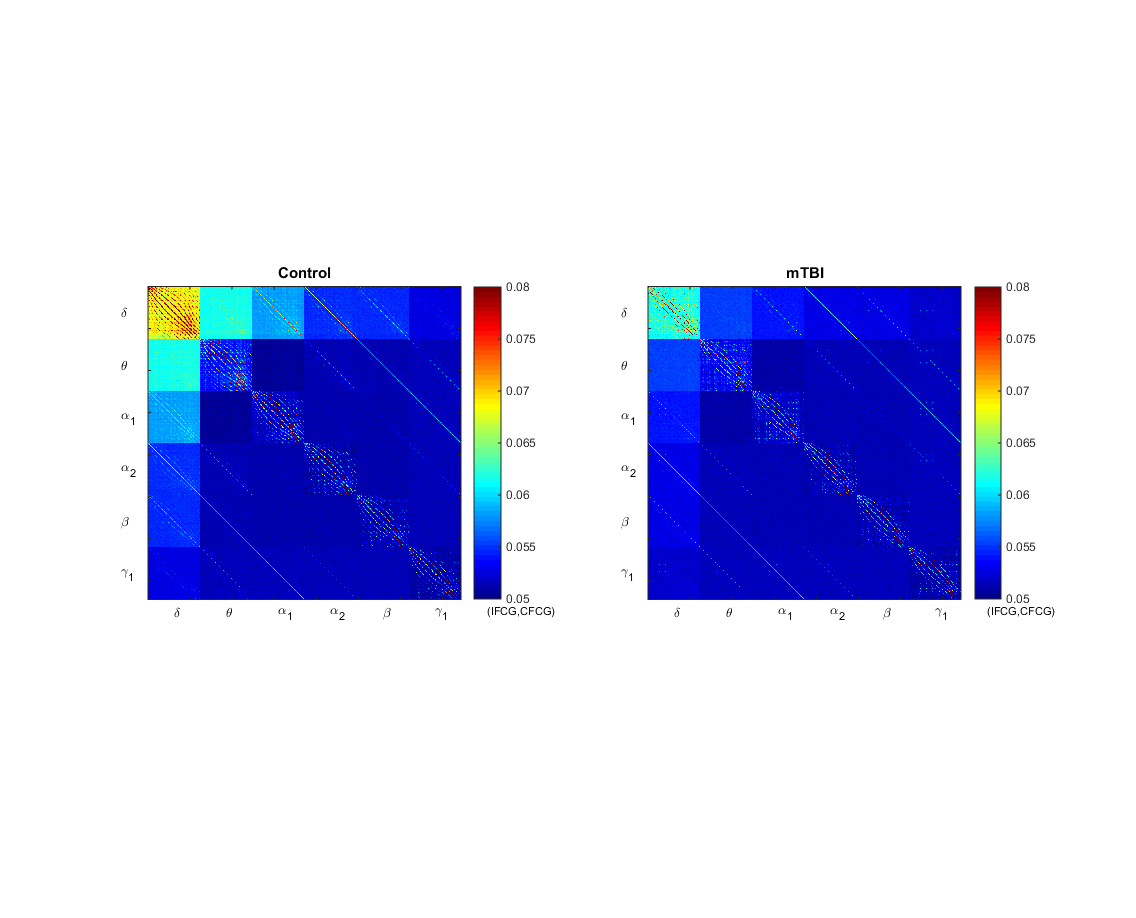


**Figure S2.** Graphical representation of the mean value of a multi-layer graph for Normal Control and mTBI groups. The diagonal cells correspond to the IFCG whereas the off-diagonal cells correspond to the CFCG.

The dimensions of the matrices presented to the classification scheme were (subjects x bands x frequency pairs x number of sensors) for each group. The results from the classification scheme of “TSA+kNN” (for k = 10) are presented in Table S1. These results demonstrate that the multi-layer graphs of the mTBI group can be separated with high accuracy from the graphs of the normal control group. In contrast to the classification scheme of the ICFCG, the current scheme requires a longer execution time, and the final results appear lower classification performance.

**Table S1**: Classification performance of the multi-layer graph after 10-fold cross-validation using TSA for feature selection and kNN for classification.

| Classification Scheme | Accuracy | Sensitivity | Specificity |
| --- | --- | --- | --- |
| TSA+kNN | 89.5±1.6 | 92.4±3.2 | 81±7.2 |

1. **Estimation of Small World and Rich Club Network Organizations**

The SW network reached a smaller Global Efficiency (GE) value than a random network, while its Local Efficiency (LE) was higher compared to a random network. The calculation of the randomized GE and LE, GE_RAND_ and LE_RAND_, respectively, for the random network was based on a permutation procedure (Dimitriadis et al., 2015b) that preserved the out-strength but not the in-strength distribution. The procedure was repeated 1000 times and averaged across all random networks to obtain GE_RAND_ and LE_RAND_. The dimensions of GE_RAND_ and LE_RAND_ were 248 x 1000. Two indices γ = LE/<LE_RAND_> and λ = GE/<GE_RAND_> were then calculated for the FCG under study and the ratio S_ratio_ = γ/λ was derived with dimensions 1 x 248. This ratio was greater than 1 in SW networks (Dimitriadis et al., 2015a; Antonakakis et al., 2015). SW nodes were detected using S_r_ > S_r_^avg^ + S_r_^SD^ where S_r_^avg^ was the average value and S_r_^SD^ was the standard deviation across all sensors.

To estimate the RC nodes for all types of FCG, we first computed the degree k of each node on each FCG. For each value of k, the subset of nodes with degree larger than k was selected. Using this subset, which consisted of n nodes and the corresponding E_>k_ connections, the total sum of weights W_>k_ was determined for all edges (van den Heuvel et al, 2011). The weighted RC parameter, Φ_w_, was then computed for each degree k and the total weight W_>k_ was normalized over the sum of the weights of the strongest E_>k_ connections of the network (van den Heuvel et al, 2011). The estimation of Φ_w_ was performed using the Brain Connectivity Toolbox^[[2]](#footnote-2)^** (Rubinov and Sporns, 2010). In our analysis, 1000 random networks preserving the degree distribution and sequence of the original network (van den Heuvel et al, 2011) were generated, and the RC coefficient was computed for each random network and degree k. Φ^r^_w_ was computed as the average RC coefficient over the random networks and the normalized RC parameter Φ^n^_w_ was computed as the ratio of Φ_w_ to Φ^r^_w_. A network followed an RC organization if Φ^n^_w_ > 1, for a continuous range of k. The randomization process was used to assess the statistical significance of the results through permutation testing (van den Heuvel et al, 2011). To this respect, the distribution of Φ^r^_w_ yielded the null distribution of RC coefficients obtained from random topologies. Using this null distribution, Φ_w_ was assigned a p-value from the percentage of random tests found to be more extreme than the observed RC coefficient Φ_w_. All tests were conducted controlling for the false discovery rate (FDR) (Benjamini and Hochberg, 1995).

1. **Differences on network properties per lobe**

Using parametric and non-parametric statistical analysis (Antonakakis et al., 2013; 2016), we explored statistical differences on the distribution of the rich-club (RC) organization between the two groups for FCG type and each lobe, namely left frontal (lf), left temporal (lt), left occipital (lo), left parietal (lp), the corresponding right sides, rf, rt, ro, rp, and centro-frontal (cf) and centro-occipital (co).

**Table S2**. Statistical analysis on the distribution of the RC organization between the two groups for each type of FCG. Shaded cells denote statistically significant differences (p<0.05). Higher probability values are shown in red for the mTBI group and blue for the control group.

| **IFCG** | | δ | | | θ | | | | | α | | | | β | | | | γ1 | | | | | γ2 | | |  |
| --- | --- | --- | --- | --- | --- | --- | --- | --- | --- | --- | --- | --- | --- | --- | --- | --- | --- | --- | --- | --- | --- | --- | --- | --- | --- | --- |
| lf | | | 0.36 | | | | 0.66 | | | | 0.57 | | | | 0.68 | | | | | 1.00 | | | | 1.00 | | |
| lo | | | 0.97 | | | | 0.39 | | | | 1.00 | | | | 1.00 | | | | | 1.00 | | | | 1.00 | | |
| lp | | | 0.02 | | | | 0.24 | | | | 0.36 | | | | 0.02 | | | | | 0.20 | | | | 0.68 | | |
| lt | | | 0.02 | | | | 0.73 | | | | 0.39 | | | | 0.57 | | | | | 0.23 | | | | 0.50 | | |
| rf | | | 0.57 | | | | 0.02 | | | | 0.02 | | | | 0.02 | | | | | 0.02 | | | | 0.16 | | |
| ro | | | 0.24 | | | | 0.36 | | | | 0.73 | | | | 1.00 | | | | | 1.00 | | | | 0.97 | | |
| rp | | | 0.16 | | | | 0.68 | | | | 0.90 | | | | 1.00 | | | | | 1.00 | | | | 0.68 | | |
| rt | | | 0.31 | | | | 0.90 | | | | 0.68 | | | | 0.34 | | | | | 1.00 | | | | 0.96 | | |
| cf | | | - | | | | - | | | | - | | | | - | | | | | - | | | | - | | |
| co | | | - | | | | - | | | | - | | | | - | | | | | - | | | | - | | |
| **CFCG** | | | (δ.β) | | | | | (δ.γ1) | | | | | (θ.β) | | | | (θ.γ1) | | | | | (β.γ2) | | | | |
| lf | | | 1.00 | | | | | 0.88 | | | | | 0.59 | | | | 0.93 | | | | | 1.00 | | | | |
| lo | | | 0.02 | | | | | 0.01 | | | | | 0.04 | | | | 0.27 | | | | | 0.04 | | | | |
| lp | | | 0.20 | | | | | 1.00 | | | | | 0.04 | | | | 1.00 | | | | | 0.12 | | | | |
| lt | | | 0.58 | | | | | 0.04 | | | | | 0.07 | | | | 0.02 | | | | | 0.12 | | | | |
| rf | | | 0.75 | | | | | 1.00 | | | | | 1.00 | | | | 0.44 | | | | | 1.00 | | | | |
| ro | | | 0.58 | | | | | 0.40 | | | | | 1.00 | | | | 0.42 | | | | | 0.24 | | | | |
| rp | | | 0.90 | | | | | 1.00 | | | | | 0.33 | | | | 0.88 | | | | | 0.02 | | | | |
| rt | | | 0.85 | | | | | 0.24 | | | | | 0.12 | | | | 0.27 | | | | | 0.12 | | | | |
| cf | | | - | | | | | - | | | | | - | | | | - | | | | | - | | | | |
| co | | | - | | | | | - | | | | | - | | | | - | | | | | - | | | | |
| **ICFCG** |  | | |  | |  | | |  | | |  | | | |  | | |  | |  | | | |  | |
| lf | lo | | | lp | | lt | | | rf | | | ro | | | | rp | | | rt | | cf | | | | co | |
| 0.82 | 0.24 | | | 0.92 | | 0.24 | | | 0.92 | | | 0.92 | | | | 0.82 | | | 0.80 | | - | | | | - | |

1. **Subject Demographics**

The current study is part of a larger mTBI project (Levin, 2009) supported by the Department of Defense (DoD). The subjects included in this analysis included a group of 30 right-handed patients with mTBI (29.33 ± 9.2 years of age) from the DoD project and a group of 50 age-matched neurologically intact controls (29.25 ± 9.1 years of age) drawn from a database that was being assembled as a normative data repository at UTHSC-Houston. The definition of mTBI used followed the guidelines of DoD (Assistant Secretary, 2007) and the American Congress of Rehabilitation Medicine (Kay et al., 1993). mTBI subjects were recruited from the Emergency Departments (EDs) of two Level 1 trauma centers and one Level III community hospital in a large ethnically diverse southwestern metropolitan area. Subjects were recruited by healthcare professionals (RN, MD, EMT-P) who had clinical experience with brain injury patients, knowledge of research, and excellent interpersonal and problem-solving skills. Screening occurred through review of data in the EDs electronic healthcare system (EHS), consultation with ED staff, and subject interviews. Special permission was obtained from the institutional IRBs to administer the Galveston Orientation and Amnesia Test (GOAT) (Levin et al., 2008) prior to obtaining informed consent to identify cognitive impairment that would preclude provision of informed consent. All subjects showed GOAT scores of 75 or greater and so have provided informed consent.

Inclusion criteria for the mTBI subjects included age 18-50 years, injury occurring within the preceding 24 hours, presence of a head injury (documented in medical records and/or verified by witnesses), Glasgow Coma Scale (GCS) (Teasdale & Jennett, 1974) score 13-15, loss of consciousness <30 minutes including 0 minutes, post-traumatic amnesia <24 hours including 0 minutes, and a negative head computed tomography (CT) scan. Exclusion criteria included a score on the Abbreviated Injury Scale (AIS) >3 for any body part, history of significant pre-existing disease (e.g., psychotic disorder, bipolar disorder, post-traumatic stress disorder (PTSD) diagnosed by a psychiatrist or psychologist, past treatment for alcohol dependence or substance abuse), blood alcohol level >80 mg/dL at the time of consent, documentation of intoxication, left-handedness, and contraindications for MRI (including claustrophobia and pregnancy). Previous head injury requiring hospitalization or ED treatment was also an exclusion criterion. The demographics of mTBI subjects and the location of injury are given in Table S3.

The normative data repository included neurologically intact right-handed adults recruited from the University of Texas Medical School (UTMS) population (medical students and fellows). Handedness was assessed using the Edinburgh Handedness Inventory (Oldfield, 1971). Participants were screened, using self-report, for medication affecting the neurophysiological activity of the brain, as well as metallic implants, such as dental crowns, which affect the MEG evoked fields. Previous head injury, history of neurological or psychiatric disorder, substance abuse, and extensive dental work and implants incompatible with MEG were exclusion criteria for the control subjects. The project was approved by the Institutional Review Boards at the participating institutions and the Human Research Protection Officials review of research protocols for DoD. All procedures were compliant with the Health Insurance Portability and Accountability Act (HIPAA).

**Table S3**. Subject demographics, location, and mode of impact (MOI) for the mTBI group.

| **Subject ID** | **Age at injury** | **Gender** | **Primary MOI** | **Primary MOI Type** | **Primary MOI Location** |
| --- | --- | --- | --- | --- | --- |
| 1 | 21.7 | M | Auto Pedestrian | Laceration - no sutures | Head |
| 2 | 22.1 | M | Motor Vehicle | Tenderness | Head |
| 3 | 43.1 | M | Motor Vehicle | Tenderness | Head |
| 4 | 34.6 | M | Fall Raised Surface | Abrasion | Head |
| 5 | 42.3 | F | Assault | Bruising | Head |
| 6 | 20.3 | M | Motor Vehicle | Bruising | Head |
| 7 | 24.0 | F | ATV | Laceration - no sutures | Head |
| 8 | 24.9 | M | Sports-related | Laceration - with sutures | Head |
| 9 | 24.4 | F | Motor Vehicle | Bruising | Head/Face |
| 10 | 43.7 | F | Motor Vehicle | Tenderness | Head |
| 11 | 36.3 | M | Blow to Head | Tenderness | Head |
| 12 | 49.1 | M | Motorcycle | Contusion | Head |
| 13 | 43.3 | F | Fall Standing | Laceration - no sutures | Head |
| 14 | 23.3 | F | Fall Standing | Laceration - with sutures | Head |
| 15 | 33.4 | M | Fall Raised Surface | Laceration - no sutures | Head |
| 16 | 27.3 | M | Auto Pedestrian | Tenderness | Head/Face |
| 17 | 49.8 | F | Fall Moving Object | Laceration - with sutures | Head |
| 18 | 25.3 | M | Fall | Abrasion | Head |
| 19 | 27.7 | M | Fall Moving Object | Abrasion | Head |
| 20 | 20.5 | M | Motor Vehicle | Bruising | Head |
| 21 | 27.0 | F | Auto Pedestrian | Bruising | Head |
| 22 | 22.6 | F | Motor Vehicle | Contusion | Head |
| 23 | 34.8 | M | Assault | Contusion | Head |
| 24 | 20.3 | M | Sports-related | Contusion | Head/Face |
| 25 | 43.8 | F | Fall Standing | Contusion | Head |
| 26 | 28.8 | F | Motor Vehicle | Contusion | Head |
| 27 | 27.8 | M | Assault | Contusion | Head |
| 28 | 24.7 | F | Assault | Contusion | Head |
| 29 | 22.8 | F | Assault | Contusion | Head |
| 30 | 19.3 | M | Assault | Contusion | Head |

1. **References**

Antonakakis M, Dimitriadis SI, Zervakis M, Rezaie R, Babajani-Feremi A, Micheloyannis S, Gouridakis G, Papanicolaou AC (2015) Comparison of brain network models using cross-frequency coupling and attack strategies. Conf Proc IEEE Eng Med Biol Soc, 7426–7429.

Antonakakis M., Dimitriadis S.I., Zervakis M., Micheloyannis S., Rezaie R., Babajani-Feremi A., Zouridakis G., Papanicolaou A.C., (2016). Altered cross-frequency coupling in resting-state MEG after mild traumatic brain injury. Int J Psychophysiol. 102, 1–11.

Antonakakis, M., Giannakakis, G., Tsiknakis, M., Micheloyannis, S., & Zervakis, M. (2013). Synchronization coupling investigation using ICA cluster analysis in resting MEG signals in reading difficulties. In *2013 IEEE 13th International Conference on Bioinformatics and Bioengineering (BIBE)* (pp. 1–5). <http://doi.org/10.1109/BIBE.2013.6701594>

Aru, J., Aru, J., Priesemann, V., Wibral, M., Lana, L., Pipa, G., et al. (2015). Untangling cross-frequency coupling in neuroscience. *Curr. Opin. Neurobiol.* 31, 51–61. doi: 10.1016/j.conb.2014.08.002

Assistant Secretary, D.o.D., 10–1-2007. Traumatic Brain Injury: Definition and Reporting. Department of Defense.

Bassett, D.S., Bullmore, E.T., Meyer-Lindenberg, A., Apud, J.A., Weinberger, D.R., Coppola, R., (2009). Cognitive fitness of cost-efficient brain functional networks. Proc. Natl. Acad. Sci. U. S. A. 106, 11747–11752.

Benjamini Y, & Hochberg Y (1995) Controlling the False Discovery Rate: A Practical and Powerful Approach to Multiple Testing. J R Statist Soc 57:289–300.

Bullmore ET, & Bassett DS (2011) Brain graphs: graphical models of the human brain connectome. Annu Rev Clin Psychol 7:113–140.

Canolty, R. T., Edwards, E., Dalal, S. S., Soltani, M., Nagarajan, S. S., Kirsch, H. E., et al. (2006). High gamma power is phase-locked to theta oscillations in human neocortex. *Science* 313, 1626–1628. doi: 10.1126/science.1128115

Claerbout, Jon F (1985). Fundamentals of Geophysical Data Processing with Applications to Petroleum Prospecting. Oxford, UK: Blackwell, 59–62.

Delorme A, & Makeig S (2004) EEGLAB: an open source toolbox for analysis of single-trial EEG dynamics including independent component analysis. J Neurosci Methods 134:9–21.

Dimitriadis SI, Zouridakis G, Rezaie R, Babajani-Feremi A, & Papanicolaou AC (2015b) Functional connectivity changes detected with magnetoencephalography after mild traumatic brain injury. NeuroImage Clin 9:519–531.

Dimitriadis, S. I., Laskaris, N. A., & Tzelepi, A. (2013a). On the quantization of time-varying phase synchrony patterns into distinct functional connectivity microstates (FCμstates) in a multi-trial visual ERP paradigm. *Brain Topography*, *26*(3), 397–409. http://doi.org/10.1007/s10548-013-0276-z

Dimitriadis, S. I., Zouridakis, G., Rezaie, R., Babajani-Feremi, A., & Papanicolaou, A. C. (2015a). Functional connectivity changes detected with magnetoencephalography after mild traumatic brain injury. *NeuroImage: Clinical*, *9*, 519–531. <http://doi.org/10.1016/j.nicl.2015.09.011>

Duda, R. O., Hart, P. E., & Stork, D. G. (2012). Pattern classification. John Wiley & Sons.

Escudero J, Hornero R, Abásolo D, & Fernández A (2011) Quantitative evaluation of artifact removal in real magnetoencephalogram signals with blind source separation. Ann Biomed Eng 39:2274–2286.

He X, Cai D, Niyogi P. Laplacian score for feature selection. In Advances in Neural Information Processing Systems 18 Weiss, Scholkopf, Platt (editors). MIT Press, Cambridge, MA, 2005.

Kay T (1993) Mild traumatic brain injury committee of the head injury interdisciplinary special interest group of the American Congress of Rehabilitation Medicine. Definition of mild traumatic brain injury. J Head Trauma Rehabil 8:86–87.

Levin HS (2009) Mission Connect Mild TBI Translational Research Consortium. Baylor College of Medicine Houston TX.

Levin HS, O'Donnell VM, & Grossman RG. (2008). The Galveston Orientation and Amnesia Test: A practical scale to assess cognition after head injury. J Nerv Ment Dis 167, 675-684.

Oldfield, R. C. (1971). The assessment and analysis of handedness: the Edinburgh inventory. Neuropsychologia, 9(1), 97-113.

Oostenveld R, Fries P, Maris E, Schoffelen, (2010) FieldTrip: Open Source Software for Advanced Analysis of MEG, EEG, and Invasive Electrophysiological Data. Comput Intell Neurosci 2011:e156869.

Pollonini L., Patidar U., Situ N., Rezaie R., Papanicolaou A.C., Zouridakis G. Functional connectivity networks in the autistic and healthy brain assessed using Granger causality. Conf. Proc. I.E.E.E. Eng. Med. Biol. Soc. 2010; 1730–1733. [21096408](http://www.ncbi.nlm.nih.gov/pubmed/21096408) [[PubMed](http://www.ncbi.nlm.nih.gov/pubmed/21096408)]

Richiardi, J., Eryilmaz, H., Schwartz, S., Vuilleumier, P., Van De Ville D., 2011. Decoding brain states from fMRI connectivity graphs. Neuroimage 56(2), 616-626.

Shen, H., Wang, L., Liu, Y., Hu, D., 2010. Discriminative analysis of resting-state functional connectivity patterns of schizophrenia using low dimensional embedding of fMRI. Neuroimage 49(4), 3110-3121.

Teasdale, G.M., Jennet, B., 1974. Assessment of coma and impaired consciousness. Lancet 81– 84. Teasdale, G.M., Jennet, B., 1974. Assessment of coma and impaired consciousness. Lancet 81– 84.

Theiler, J., Eubank, S., Longtin, A., Galdrikian, B., and Farmer, J. D. (1992). Testing for nonlineaity in time series: the method of surrogate data. *Physica D* 85:77. doi: 10.1016/0167-2789(92)90102-S

Tsiaras V, Simos PG, Rezaie R, Sheth BR, Garyfallidis E, Castillo EM, & Papanicolaou AC (2011) Extracting biomarkers of autism from MEG resting-state functional connectivity networks. Comput Biol Med 41:1166–1177.

Van den Heuvel MP, & Sporns O (2011) Rich-Club Organization of the Human Connectome. J Neurosci 31:15775–15786.

Voytek, B., Canolty, R. T., Shestyuk, A., Crone, N. E., Parvizi, J., & Knight, R. T. (2010). Shifts in Gamma Phase–Amplitude Coupling Frequency from Theta to Alpha Over Posterior Cortex During Visual Tasks. Frontiers in Human Neuroscience, 4. http://doi.org/10.3389/fnhum.2010.00191

1. <http://users.auth.gr/~stdimitr/software.html> - I would appreciate it if you refer to the toolbox and the paper Dimitriadis et al., 2015b. [↑](#footnote-ref-1)
2. ** https://sites.google.com/site/bctnet/ [↑](#footnote-ref-2)
